# The comparative evidence of efficacy of non-invasive brain and nerve stimulation in diabetic neuropathy: a systematic review and network meta-analysis

**DOI:** 10.1186/s12984-025-01614-y

**Published:** 2025-04-19

**Authors:** Ping-Tao Tseng, Bing-Yan Zeng, Chih-Wei Hsu, Chao-Ming Hung, Brendon Stubbs, Yen-Wen Chen, Tien-Yu Chen, Jiann-Jy Chen, Wei-Te Lei, Yow-Ling Shiue, Chih-Sung Liang

**Affiliations:** 1 Institute of Precision Medicine, National Sun Yat-Sen University, No. 70, Lienhai Road, Kaohsiung, 80424 Taiwan; 2 Institute of Biomedical Sciences, National Sun Yat-Sen University, Kaohsiung, Taiwan; 3 Department of Psychology, College of Medical and Health Science, Asia University, Taichung, Taiwan; 4 Prospect Clinic for Otorhinolaryngology & Neurology, No. 252, Nanzixin Road, Nanzi District, Kaohsiung, 81166 Taiwan; 5 Department of Internal Medicine, E-Da Dachang Hospital, I-Shou University, Kaohsiung, Taiwan; 6 Department of Psychiatry, Kaohsiung Chang Gung Memorial Hospital and Chang Gung University College of Medicine, Kaohsiung, Taiwan; 7 Division of General Surgery, Department of Surgery, E-Da Cancer Hospital, I-Shou University, Kaohsiung, Taiwan; 8 School of Medicine, College of Medicine, I-Shou University, Kaohsiung, Taiwan; 9 Department of Psychological Medicine, Institute of Psychiatry, Psychology and Neuroscience, King’s College London, London, UK; 10 Department of Sport Science, University of Vienna, Vienna, Austria; 11 Department of Psychiatry, Beitou Branch Tri-Service General Hospital; School of Medicine, National Defense Medical Center, Beitou District, No. 60, Xinmin Road, Taipei, 112 Taiwan; 12 Institute of Brain Science, National Yang Ming Chiao Tung University, Taipei, 112 Taiwan; 13 Department of Otorhinolaryngology, E-Da Cancer Hospital, I-Shou University, Kaohsiung, Taiwan; 14 Section of Immunology, Rheumatology, and Allergy Department of Pediatrics, Hsinchu Municipal Mackay Children’S Hospital, No. 690, Section 2, Guangfu Road, East District, Hsinchu, 300044 Taiwan; 15 Center for Molecular and Clinical Immunology, Chang Gung University, Taoyuan, Taiwan; 16 Department of Psychiatry, Beitou Branch, Tri-Service General Hospital; School of Medicine, National Defense Medical Center, Taipei, Taiwan; 17 Department of Psychiatry, National Defense Medical Center, Taipei, Taiwan; 18 Department of Medicine, MacKay Medical College, New Taipei, 25245 Taiwan

**Keywords:** Network meta-analysis, TENS, Transcutaneous electrical nerve stimulation, Non-invasive brain stimulation, Non-invasive nerve stimulation, Diabetes

## Abstract

**Background:**

Diabetes mellitus is a highly burdensome metabolic disorder, affecting over 100 million people worldwide and leading to numerous complications. Among these, diabetic neuropathy is one of the most common, with approximately 60% of individuals with diabetes developing this condition. Current pharmacological treatments for diabetic neuropathy are often inadequate, providing limited efficacy and accompanied by a range of adverse effects. Non-invasive brain and nerve stimulation techniques have been proposed as potentially beneficial for diabetic neuropathy, though existing evidence remains inconclusive. This systematic review and network meta-analysis (NMA) aimed to evaluate the comparative efficacy of various non-invasive brain and nerve stimulation interventions in patients with diabetic neuropathy.

**Methods:**

A systematic search of electronic databases was conducted to identify randomized controlled trials (RCTs) of non-invasive brain or nerve stimulation in patients with diabetic neuropathy, from inception to September 6, 2024. The primary outcome was the change in pain severity, while secondary outcomes included changes in quality of life and sleep disturbance. Acceptability was assessed through dropout rates (i.e., withdrawal from the study before completion for any reason). A frequentist-based NMA was performed, utilizing odds ratios (OR) and standardized mean differences (SMD) with 95% confidence intervals (95%CIs) as effect size measures.

**Results:**

The NMA, which included 15 RCTs (totaling 1,139 participants, with a mean age of 61.2 years and a mean female proportion of 53.8%), evaluated 10 experimental interventions (1 control group, 4 non-invasive brain stimulation methods, and 5 non-invasive nerve stimulation methods). The analysis revealed that only transcutaneous electrical nerve stimulation (TENS) was associated with significantly greater improvements in pain severity (SMD = − 1.67, 95%CIs = − 2.64 to − 0.71) and sleep disruption (SMD = − 1.63, 95%CIs = − 2.27 to − 0.99) compared to the control group. None of the studied interventions showed significant differences in dropout rates or all-cause mortality compared to the control group.

**Conclusion:**

This study provides comparative evidence supporting the use of specific brain and nerve stimulation interventions in managing diabetic neuropathy. Future well-designed RCTs with longer treatment durations are recommended to further validate the long-term efficacy of these interventions.

*Trial registration* PROSPERO CRD42024587660.

**Supplementary Information:**

The online version contains supplementary material available at 10.1186/s12984-025-01614-y.

## Introduction

Diabetes mellitus is a highly burdensome metabolic disorder, affecting over 100 million individuals globally [1]. It is associated with a substantial mortality rate of 18.5 per 100,000 population and a disability-adjusted life year (DALY) rate of 801.5 per 100,000 population, primarily due to its numerous complications [2]. Among these complications, diabetic neuropathy is one of the most prevalent, affecting approximately 60% of patients with diabetes mellitus [1]. Of those, 43% to 53% experience painful symptoms in their extremities [3].

Despite its high prevalence, effective treatment for diabetic neuropathy remains limited and challenging [4]. Simple symptomatic pharmacotherapy could provide limited efficacy in restoring damaged nerves or their function [1]. Further, many of these treatments are associated with undesirable side effects. On the other hand, researchers noticed that, in animal model, low intensity electrical stimulation could promote nerve regeneration after nerve injury [5].

To address this clinical challenge, researchers have explored the use of non-invasive brain and nerve stimulation techniques for managing diabetic neuropathy. These methods include brain stimulation, commonly referred to as neurostimulation or neuromodulation, which works by inducing an electric or magnetic field in targeted brain regions [6]. By adjusting stimulation parameters, these techniques can amplify or suppress neuronal activity [7]. Similarly, nerve stimulation—administered through electrical or magnetic methods—functions by indirectly stimulating endogenous opioids at the spinal cord level [8] or by improving endoneurial blood flow and restoring nerve conduction velocity [9].

Building on the theoretical benefits of non-invasive brain and nerve stimulation in improving outcomes for diabetic neuropathy, several new modalities have been developed. These include brain stimulation techniques such as transcranial direct current stimulation (tDCS), deep transcranial magnetic stimulation (dTMS), and repetitive transcranial magnetic stimulation (rTMS), as well as nerve stimulation methods like frequency-modulated electromagnetic neural stimulation (FREMS), pulsed electromagnetic field (PEMF), static electromagnetic field (SEMF), and transcutaneous electrical nerve stimulation (TENS). These modalities have demonstrated not only promising efficacy but also an acceptable safety profile in terms of dropout rates and all-cause mortality [6].

Multiple randomized controlled trials (RCTs) have been conducted to assess the efficacy of these non-invasive brain and nerve stimulation methods for managing diabetic neuropathy. Some traditional pairwise meta-analyses have summarized the available evidence [10, 11]; however, the results have been inconsistent. Furthermore, traditional pairwise meta-analyses are unable to provide detailed comparisons between the different non-invasive brain and nerve stimulation methods.

Given this context, a well-designed network meta-analysis (NMA) offers the advantage of estimating comparative efficacy and understanding the relative merits of different interventions. Based on its methodological superiority, NMA could provide more comprehensive evidence to assist decision making process in either daily medical practice [12] or in psychological approach [13] than the traditional pair-wise meta-analyses did. A well-designed NMA, when used appropriately, could help in health promotion [14] so that it might ultimately lead to improvement in overall social economics. To the best of our knowledge, no NMAs have been conducted to evaluate the efficacy of various non-invasive brain and nerve stimulation techniques in patients with diabetic neuropathy. Therefore, the aim of this systematic review and NMA is to compare the efficacy of different non-invasive brain and nerve stimulation methods in the management of diabetic neuropathy.

## Methods

This network meta-analysis (NMA) adhered to the guidelines outlined in the Preferred Reporting Items for Systematic Reviews and Meta-Analyses (PRISMA) extension for network meta-analyses (PRISMA NMA) [15] (eTable 1). The protocol was registered with PROSPERO under the registration number CRD42024587660 and received approval from the Institutional Review Board of the Tri-Service General Hospital, National Defense Medical Center, Taipei, Taiwan (IRB No. B- 109–29).

### Database searches and study identification

Comprehensive searches were conducted across multiple databases, including PubMed, Embase, ClinicalKey, Cochrane CENTRAL, ProQuest, ScienceDirect, Web of Science, and ClinicalTrials.gov. The search for eligible studies began on September 6, 2024. The search term was (transcutaneous electrical nerve stimulator OR TENS OR pulsed electromagnetic field OR PEMF OR deep transcranial magnetic stimulation OR dTMS OR repetitive transcranial magnetic stimulation OR rTMS OR TMS OR non-invasive brain stimulation OR non-invasive nerve stimulation OR theta burst stimulation OR transcranial direct current stimulation OR TBS OR tDCS OR vagus nerve stimulation OR vagal nerve stimulation OR tVNS OR nVNS OR VNS OR static magnetic field stimulation OR colon electric stimulation) AND (diabetic neuropathy OR diabetic polyneuropathy) AND (random OR randomized OR randomised) in the PubMed. However, since the search syntax and search logic varied across databases, we listed the detailed search term and search result in eTable 2. Two independent reviewers (PT Tseng and BY Zeng) conducted the electronic searches, and screened titles and abstracts. In cases of discrepancy, a third reviewer (CS Liang) would be consulted and finally achieved a resolution through consensus. Additionally, reference lists from review articles were manually screened for relevant studies [6, 16–18]. No language restrictions were applied to the search.

### Inclusion and exclusion criteria

The NMA followed the PICOS framework (Population, Intervention, Comparison, Outcome, Study design) with the following criteria: (1) Population: human patients with diabetic neuropathy; (2) Intervention: non-invasive brain or nerve stimulation; (3) Comparison: control group, including either standard care or sham control; (4) Outcome: changes in pain severity; and (5) Study: randomized controlled trials (RCTs). To limit heterogeneity, only trials investigating non-invasive brain or nerve stimulation interventions were included. Trials involving a single stimulation session were excluded, as these interventions are designed to be efficacious across an entire treatment course.

For inclusion, studies were required to: (1) recruit patients with diabetic neuropathy; (2) evaluate the efficacy of non-invasive brain or nerve stimulation interventions; and (3) be conducted in humans.

Exclusion criteria included: (1) non-RCTs; (2) RCTs without patients with diabetic neuropathy; (3) RCTs not comparing non-invasive brain or nerve stimulation interventions; (4) RCTs not reporting target outcomes; (5) RCTs limited to a single stimulation session; and (6) animal studies.

### Methodological quality appraisal

To recognize the quality of included studies, two reviewers independently assessed the risk of bias for using the Cochrane Risk of Bias Tool 1.0 [19], achieving inter-rater reliability of 0.88. Discrepancies were resolved by a third reviewer.

### Outcome definition

The primary outcome of this NMA was the change in pain severity. As different studies used various scales to assess pain severity, no restrictions were imposed on the specific pain rating scales. Secondary outcomes included changes in quality of life and sleep disruption. Treatment acceptability was measured by the dropout rate.

### Data extraction, management and conversion

Two authors (PT Tseng and BY Zeng) independently extracted data, including demographic information, study design, treatment protocols, and both primary and secondary outcomes. In cases where necessary data were missing, corresponding authors were contacted. Data extraction followed the Cochrane Handbook for Systematic Reviews of Interventions and relevant medical literature guidelines [20].

### Statistical analyses

Given the presence of multiple treatment arms, a random-effects model was employed for the NMA [21], using MetaInsight (version 4.0.2, Complex Reviews Support Unit, National Institute for Health Research, London, UK) within a frequentist framework. MetaInsight, a web-based platform for conducting NMAs, incorporated the netmeta package in R software to perform frequentist statistical analyses [22].

Forest plots were generated for odds ratios (OR) with 95% confidence intervals (95% CIs) for continuous outcomes such as dropout rates, and standardized mean differences (SMD) with 95%CIs for categorical outcomes, including changes in pain severity, quality of life, and sleep disruption [23]. Treatments were then ranked, and effect sizes for direct and indirect comparisons were presented in tables. A"node splitting"method was used to assess consistency between direct and indirect treatment effect estimates, a process well-suited for NMAs with access to trial-level data [22, 24]. Statistical significance was set at a two-tailed p-value of less than 0.05.

### Sensitivity analyses

To evaluate the robustness of our findings, sensitivity analyses were conducted by subgrouping RCTs based on either (1) the target regions of stimulation; or (2) the duration of treatment. Specifically, stimulation methods were divided into (1) brain-targeted (e.g., TMS, tDCS) and nerve-targeted (e.g., PEMF, TENS, SEMF, FREMS) categories; or (2) short-term (less than 1 year) versus long-term (at least 1 year) treatment durations.

### General declaration

This study conforms to the provisions of the Declaration of Helsinki.

## Results

### Eligibility of the studies

**Fig. 1:**
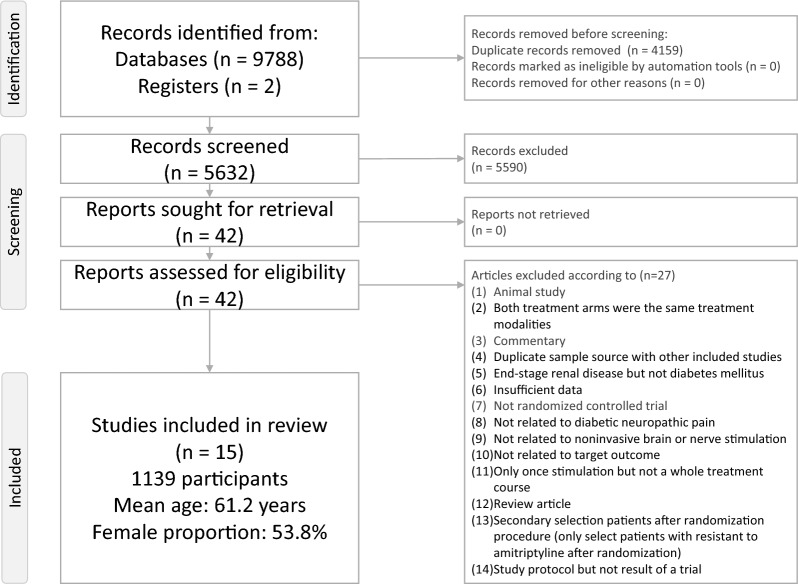
PRISMA2020 Flowchart of current network meta-analysis

Figure 1 presents the flowchart detailing the literature search and screening process for this NMA. A total of 27 articles were excluded for various reasons (eTable 3), leaving 15 RCTs for inclusion (Table 1) [25–39]. These studies involved 1,139 participants (mean age = 61.2 years, range: 54.5 to 70.6 years; mean female proportion = 53.8%, range: 39.1% to 66.3%). The average treatment duration was 10.3 weeks (range: 1 to 16 weeks), while the mean study duration, including post-treatment follow-up, was 11.0 weeks (range: 1 to 16 weeks). In total, 10 experimental arms were analyzed (1 control arm, 4 non-invasive brain stimulation interventions, and 5 non-invasive nerve stimulation interventions).

**Table 1:** Characteristics of the included studies

Study name	Study design	Baseline illness	Method of stimulation	Comparison	Subjects	Mean age (year)	Female (%)	Primary/secondary outcome and result	Treatment duration	Study duration	Country
Tassone, E.E. (2023) [35]	Randomized, sham-controlled, double-blind trial	Patients with diabetic peripheral neuropathy	PEMF	Pulsed Electromagnetic Fields Sham	92 90	62.3 ± 10.2 62.2 ± 9.3	54.3 53.3	Patients in the active arm experienced a clinically significant 30% reduction in pain from baseline compared to sham	16 weeks	16 weeks	USA
Yang, S. (2022) [39]	Randomized double-blind, sham-controlled trial	Patients with diabetic neuropathic pain	TMS	10 Hz rTMS over left motor cortex (C3) Sham	10 10	60.0 ± 5.0 60.8 ± 5.0	40.0 50.0	The pain intensity at 1-day and 1-week posttreatment was significantly lower than that at pretreatment	1 week	2 weeks	Korea
Ferreira, G. (2020) [27]	Sham, randomized, sham-controlled, double-blind trial	Patients with diabetic polyneuropathy	tDCS	M1 tDCS Sham	10 10	60.9 ± 15.3 60.7 ± 9.2	40.0 50.0	Short Form 36 Health Survey score increased throughout the protocol, but no difference between groups were found	1 week	3 weeks	Brazil
Bosi, E. (2013) [25]	Double-blind, randomised, multicentre, sham-controlled trial	Patients with symptomatic diabetic polyneuropathy	FREMS	Frequency-modulated electromagnetic neural stimulation Sham	50 51	59.0 ± 10.6 61.3 ± 8.3	56.0 76.5	changes in nerve conduction velocity of the three examined nerves were not different between Frequency-modulated electromagnetic neural stimulation and sham	12 weeks	12 weeks	Multiple countries
Kim, Y.J. (2013) [31]	Single-center, randomized double-blind, sham-controlled trial	Patients with diabetic chronic drug-resistant neuropathic pain	tDCS	M1 tDCS DLPFC tDCS Sham	20 20 20	59.6 ± 13.2 63.5 ± 8.8 61.6 ± 10.3	55.0 60.0 60.0	tDCS M1 group showed significantly greater reduction in pain and pain threshold versus the sham and tDCS DLPFC groups	1 week	4 weeks	Korea
Onesti, E. (2013) [33]	Single-centre, randomized, double-blind, sham-controlled trial	Patients with diabetic symmetric polyneuropathy	TMS	Deep rTMS Sham	11 12	70.7 ± 9.5 70.6 ± 7.9	36.4 41.7	Pain severity significantly decreased after real rTMS but not sham	1 week	4 weeks	Italy
Gossrau, G. (2011) [29]	Sham-controlled, single-blinded, and randomized trial	Patients with symptomatic diabetic neuropathy	TENS	Transcutaneous electric nerve stimulation Sham	21 19	67.9 ± 12.1 66.0 ± 7.1	NA	After 4 weeks of treatment, 6/21 patients in the active group vs 10/19 patients in the sham group responded to therapy	4 weeks	8 weeks	Germany
Weintraub, M.I. (2009) [36]	Randomized, double-blind, sham-controlled study	Patients with diabetic peripheral neuropathy	PEMF	Pulsed Electromagnetic Fields Sham	90 104	61.1 ± 10.4 60.6 ± 12.4	56.7 55.8	A trend toward reductions in diabetic peripheral neuropathy symptoms, favoring the Pulsed Electromagnetic Fields group	12 weeks	12 weeks	USA
Wrobel, M.P. (2008) [38]	Randomized, sham-controlled, double-blind trial	Patients with symptomatic diabetic polyneuropathy	PEMF	Pulsed Electromagnetic Fields Sham	32 29	53.6 ± 13.6 55.5 ± 10.4	62.5 55.2	Significant reductions in pain intensity were seen in both the study and control group	3 weeks	5 weeks	Poland
Bosi, E. (2005) [26]	Randomised, double-blind, Sham-controlled trial	Patients with symptomatic diabetic neuropathy	FREMS	Frequency-modulated electromagnetic neural stimulation Sham	19 19	59.2 ± 3.1 63.1 ± 3.1	NA	Frequency-modulated electromagnetic neural stimulation induced a significant reduction in daytime and night-time pain score	2 weeks	3 weeks	Italy
Reichstein, L. (2005) [34]	Randomized, open-label trial	Patients with symptomatic diabetic polyneuropathy	TENS	Transcutaneous electric nerve stimulation High-frequency external muscle stimulation	21 20	57.8 ± 12.5 64.2 ± 12.7	52.4 40.0	The responder rate was significantly higher in the high frequency group than in the TENS group	3 days	5 days	Germany
Forst, T. (2004) [28]	Double-blind, randomized, sham-controlled trial	Patients with symptomatic diabetic neuropathy	TENS	Transcutaneous electrotherapy Sham	12 7	57.6 ± 11.5 59.4 ± 8.6	50.0 42.9	Active TENS-treatment resulted in a significant improvement in total symptom score after 6 wk (− 42%) and after 12 wk (− 32%) of treatment	12 weeks	12 weeks	Germany
Weintraub, M.I. (2003) [37]	Randomized, sham-controlled, double-blind trial	Patients with symptomatic diabetic neuropathy	SEMF	Static magnetic field therapy Sham	141 118	62.6 ± 11.3 63.2 ± 11.2	46.8 49.2	There were significant reductions in burning, numbness and tingling, and exercise-induced foot pain in active group	16 weeks	16 weeks	USA
Hamza, M.A. (2000) [30]	Randomized, single-blind, sham-controlled trial	Patients with symptomatic diabetic neuropathy	TENS	Percutaneous electrical nerve stimulation Sham	25 25	56.0 ± 8.0 54.0 ± 9.0	NA	Compared with the pain scores before active and sham treatments, pain scores after treatment were reduced significantly in active but not sham group	3 weeks	4 weeks	USA
Kumar, D. (1997) [32]	Randomized, single-blind, sham-controlled trial	Patients with diabetic peripheral neuropathy	TENS	Transcutaneous electrotherapy Sham	18 13	53.0 ± 4.0 59.0 ± 3.0	61.1 61.5	In the electrotherapy group, the post-treatment pain scores were considerably lower, indicating a substantial treatment effect over and above any placebo influence	4 weeks	8 weeks	USA

Study duration, treatment duration + post-treatment follow-up duration

FREMS: frequency-modulated electromagnetic neural stimulation; NA: not available; PEMF: pulsed electromagnetic fields; SEMF: static electromagnetic field; tDCS: transcranial direct current stimulation; TENS: transcutaneous electrical nerve stimulation; TMS: transcranial magnetic stimulation

### Primary outcome: changes of pain severity

**Fig. 2:**
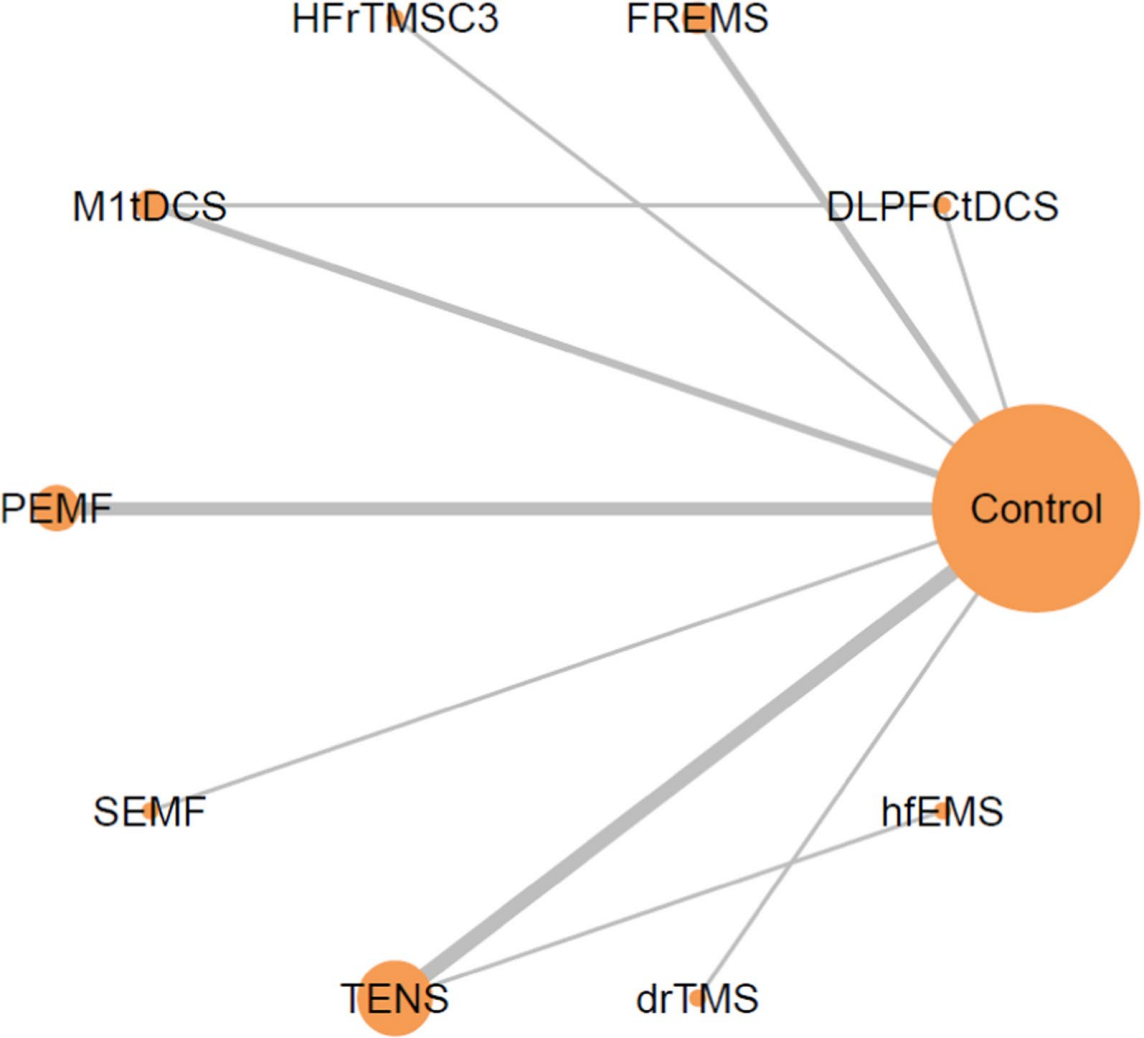
Network structure of the primary outcome: changes of pain severity. Overall structure of the network meta-analysis. The lines between nodes represent direct comparisons in various trials, and the size of each circle is proportional to the number of participants in each specific treatment. The thickness of the lines is proportional to the number of trials connected to the network

**Fig. 3:**
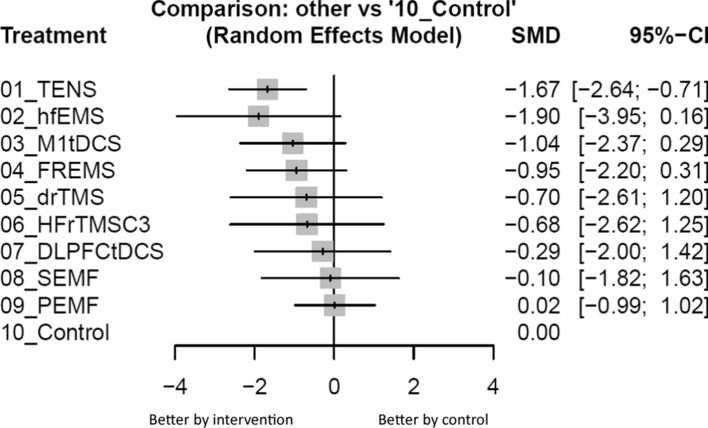
Forest plot of primary outcome: changes of pain severity. When the effect size (expressed as standardized mean differences) was less than zero, the specified treatment was associated with greater improvement in pain severity in patients with diabetic neuropathy than in patients in control groups. 95%CIs: 95% confidence intervals; DLPFCtDCS: anodal over F3 and cathodal over Fp2; drTMS: deep rTMS over bilateral parietal lobe; FREMS: frequency-modulated electromagnetic neural stimulation; hfEMS: high-frequency external muscle stimulation; HFrTMSC3: high frequency rTMS over C3; M1 tDCS: anodal over C3 and cathodal over Fp2; NA: not available; NMA: network meta-analysis; OR: odds ratio; PEMF: pulsed electromagnetic fields; RCT: randomized controlled trial; rTMS: repetitive transcranial magnetic stimulation; SEMF: static electromagnetic field; SMD: standardized mean difference; tDCS: transcranial direct current stimulation; TENS: transcutaneous electrical nerve stimulation

Only TENS (SMD = − 1.67, 95%CIs = − 2.64 to − 0.71) was associated with a significantly more reduction in pain severity than the control group. Among these interventions, TENS ranked the best intervention (Figs. 2, 3, and Table 2).

**Table 2:** League table of the primary outcome: changes of pain severity

*TENS*	0.22 [− 1.59; 2.04]								*− 1.67 [− 2.64; − 0.71]
0.22 [− 1.59; 2.04]	*hfEMS*								
− 0.63 [− 2.28; 1.01]	− 0.85 [− 3.30; 1.60]	*M1 tDCS*				− 1.01 [− 2.84; 0.81]			− 1.04 [− 2.37; 0.29]
− 0.73 [− 2.31; 0.86]	− 0.95 [− 3.36; 1.46]	− 0.10 [− 1.92; 1.73]	*FREMS*						− 0.95 [− 2.20; 0.31]
− 0.97 [− 3.11; 1.16]	− 1.19 [− 4.00; 1.61]	− 0.34 [− 2.66; 1.98]	− 0.24 [− 2.52; 2.04]	*drTMS*					− 0.70 [− 2.61; 1.20]
− 0.99 [− 3.15; 1.17]	− 1.21 [− 4.03; 1.61]	− 0.36 [− 2.70; 1.98]	− 0.26 [− 2.57; 2.04]	− 0.02 [− 2.73; 2.69]	*HFrTMSC3*				− 0.68 [− 2.62; 1.25]
− 1.39 [− 3.35; 0.58]	− 1.61 [− 4.28; 1.07]	− 0.75 [− 2.46; 0.96]	− 0.66 [− 2.78; 1.46]	− 0.41 [− 2.97; 2.14]	− 0.39 [− 2.97; 2.18]	*DLPFCtDCS*			− 0.55 [− 2.37; 1.27]
− 1.58 [− 3.56; 0.40]	− 1.80 [− 4.49; 0.88]	− 0.95 [− 3.13; 1.23]	− 0.85 [− 2.99; 1.28]	− 0.61 [− 3.18; 1.96]	− 0.59 [− 3.18; 2.00]	− 0.19 [− 2.62; 2.23]	*SEMF*		− 0.10 [− 1.82; 1.63]
*− 1.69 [− 3.09; − 0.29]	− 1.91 [− 4.20; 0.38]	− 1.06 [− 2.73; 0.61]	− 0.96 [− 2.57; 0.65]	− 0.72 [− 2.87; 1.44]	− 0.70 [− 2.88; 1.48]	− 0.30 [− 2.29; 1.68]	− 0.11 [− 2.11; 1.89]	*PEMF*	0.02 [− 0.99; 1.02]
*− 1.67 [− 2.64; − 0.71]	− 1.90 [− 3.95; 0.16]	− 1.04 [− 2.37; 0.29]	− 0.95 [− 2.20; 0.31]	− 0.70 [− 2.61; 1.20]	− 0.68 [− 2.62; 1.25]	− 0.29 [− 2.00; 1.42]	− 0.10 [− 1.82; 1.63]	0.02 [− 0.99; 1.02]	*Control*

Data present as SMD [95%CIs]. Pairwise (upper-right portion) and network (lower-left portion) meta-analysis results are presented as estimate effect sizes for the outcome of changes of pain severity in patients with diabetic neuropathy. Interventions are reported in order of mean ranking of beneficial effect on improvement of pain severity, and outcomes are expressed as standardized mean difference (SMD) (95% confidence intervals) (95%CIs). For the pairwise meta-analyses, SMD of less than 0 indicate that the treatment specified in the row got more beneficial effect than that specified in the column. For the network meta-analysis (NMA), SMD of less than 0 indicate that the treatment specified in the column got more beneficial effect than that specified in the row. Bold results marked with * indicate statistical significance

95%CIs: 95% confidence intervals; DLPFCtDCS: anodal over F3 and cathodal over Fp2; drTMS: deep rTMS over bilateral parietal lobe; FREMS: frequency-modulated electromagnetic neural stimulation; hfEMS: high-frequency external muscle stimulation; HFrTMSC3: high frequency rTMS over C3; M1 tDCS: anodal over C3 and cathodal over Fp2; NA: not available; NMA: network meta-analysis; OR: odds ratio; PEMF: pulsed electromagnetic fields; RCT: randomized controlled trial; rTMS: repetitive transcranial magnetic stimulation; SEMF: static electromagnetic field; SMD: standardized mean difference; tDCS: transcranial direct current stimulation; TENS: transcutaneous electrical nerve stimulation

#### Sensitivity analysis of primary outcomes by subgroup analysis of non-invasive nerve or brain stimulation

The main results remained similar findings in the subgroup of non-invasive nerve stimulation method. To be specific, only TENS (SMD = − 1.68, 95%CIs = − 2.66 to − 0.70) was associated with a significantly more reduction in pain severity than the control group in the subgroup of non-invasive nerve stimulation method (eFigure 1 A, eFigure 2 A, and eTable 4 A).

However, none of the investigated non-invasive brain stimulations were associated with a significantly different changes of pain severity compared to the control group (eFigure 1B, eFigure 2B, and eTable 4B).

#### Sensitivity analysis of primary outcomes by subgroup analysis of short-term or long-term treatment duration

The main results remained similar findings in the subgroup of short-term treatment duration. To be specific, only TENS (SMD = − 2.38, 95%CIs = − 4.76 to − 0.01) was associated with a significantly more reduction in pain severity than the control group in the subgroup of short-term treatment duration (eFigure 1 C, eFigure 2 C, and eTable 4 C).

However, on the other hand, only FREMS (SMD = − 0.51, 95%CIs = − 0.91 to − 0.11) was associated with a significantly more reduction in pain severity than the control group in the subgroup of long-term treatment duration (eFigure 1D, eFigure 2D, and eTable 4D).

### Secondary outcome: changes of quality of life

Only the high frequency (10 Hz) rTMS over left motor cortex (C3) (HFrTMSC3) (SMD = − 2.16, 95%CIs = − 3.26 to − 1.06) showed a significantly greater improvement in quality of life compared to the control group did. Among these interventions, HFrTMSC3 ranked as the most effective (eFigure 1E, eFigure 2E, and eTable 4E).

### Secondary outcome: sleep disruption

Only the TENS (SMD = − 1.63, 95%CIs = − 2.27 to − 0.99) was associated with a significantly greater improvement in sleep disruption compared to the control group did. It also ranked as the most effective among the interventions (eFigure 1 F, eFigure 2 F, and eTable 4 F).

### Acceptability: drop-out rate

None of the investigated treatments were associated with a significantly different drop-out rate compared to the control group (eFigure 1G, eFigure 2G, and eTable 4G).

### Risk of bias and inconsistency

In terms of risk of bias, 81.9% (86/105 items) of the studies were classified as having a low risk of bias, 14.3% (15/105 items) had an unclear risk, and 3.8% (4/105 items) were considered to have a high risk of bias (eFigures 3 A, B). The inconsistency test, which assessed the assumption of consistency across studies, revealed no significant inconsistencies in this NMA (eTable 5).

## Discussion

To the best of our knowledge, this NMA is the first systematic attempt to compare the efficacy of various non-invasive brain and nerve stimulation therapies in patients with diabetic neuropathy. The key findings of this analysis indicate that only TENS demonstrated superior efficacy across the primary and secondary outcomes, specifically in reducing pain severity and improving sleep disruption. Furthermore, HFrTMSC3 was the sole intervention associated with a significant improvement in quality of life compared to the control group. Importantly, all investigated non-invasive brain and nerve stimulation treatments exhibited similar mortality and dropout rates compared to control groups.

A significant finding of this study is that most non-invasive brain stimulation methods (i.e., central stimulation techniques) did not show superior efficacy in patients with diabetic neuropathy. This contrasts with the findings of previous traditional pairwise meta-analyses [10], which reported beneficial effects from pooled central stimulation techniques (i.e., brain stimulation) but not from pooled peripheral stimulation techniques (i.e., nerve stimulation). This discrepancy may stem from key methodological differences. Traditional pairwise meta-analyses pooled various interventions into a single category, potentially obscuring the underlying heterogeneity among the interventions. As previous reports have shown, different non-invasive brain and nerve stimulation techniques exhibit variable efficacy across different neuropsychiatric conditions [40–44]. The strength of NMA lies in its ability to provide comparative effect sizes across multiple interventions, a level of detail unattainable through traditional pairwise meta-analyses.

Another important outcome of this study is the favorable efficacy of TENS in primary and secondary outcomes, particularly in short-term treatments. The main statistical estimates came from 5 RCTs [28–30, 32, 34], which were all well-designed randomized trials. Among them, two were double-blind designed [28, 30], which all suggested a better improvement in pain severity in TENS group than sham group. The other three RCTs, either single-blind [29, 32] or open-label [34], revealed similar findings. TENS, applied via electrodes placed on the skin over the lower extremities, stimulates peripheral nerves to alleviate diabetic neuropathy symptoms. Its advantages include ease of use, affordability, non-invasiveness, and minimal adverse effects [11]. Although the precise physiological mechanism behind TENS’s pain-relieving effects remains unclear, studies suggest that it may improve endoneurial blood flow and restore nerve conduction velocity [9]. The well-perfused peripheral nerve would be associated with good clinical response through the linkage of the increased central endogenous opioid-like substances [45]. The aforementioned central endogenous opioid-like substances could indirectly inhibit the transmission of painful stimulus C fibers [46]. While the duration of TENS therapy varied across the included RCTs, previous studies have demonstrated that its beneficial effects on diabetic neuropathy can persist for an average of 1.7 years [47], suggesting long-term efficacy. In terms of acceptability, TENS was well-tolerated, with minimal adverse effects and a dropout rate comparable to control groups in this NMA [11].

On the other hand, while FREMS showed a significant reduction in pain severity in the long-term treatment subgroup, this finding should be interpreted cautiously, as it was based on a single RCT [25]. The reliability of the FREMS in long-term treatment duration should be reappraisal by the future RCTs with long treatment duration.

## Strengths and limitations

This NMA has several strengths. First, it provides comprehensive comparative evidence on the efficacy and acceptability of different brain and nerve stimulation interventions for diabetic neuropathy, which traditional pairwise meta-analyses could not achieve. Second, we enhanced the reliability of our findings by including only RCTs, avoiding potential biases from non-RCTs and case–control studies. Third, we offered clinicians a broader understanding by analyzing various outcomes, including changes in pain severity, quality of life, sleep disruption, and acceptability (i.e., dropout rate).

However, this NMA also has limitations. First, some analyses may be underpowered due to heterogeneity in experimental arms, such as differences in stimulation target regions (i.e., brain vs. nerve stimulation). To mitigate this, we conducted subgroup analyses based on stimulation target regions. Second, our strict inclusion criteria excluded non-RCTs, resulting in some treatment comparisons being based on a single RCT, such as the improvement in quality of life in the HFrTMSC3 group compared to the sham group. Despite the positive outcomes, caution is warranted in interpreting these results. Lastly, the varied treatment durations across the included trials could introduce hidden heterogeneity. To address this, we performed subgroup analyses based on treatment duration. Although FREMS was associated with significant pain reduction, this result should be interpreted cautiously due to the inclusion of only one RCT in the long-term treatment subgroup [25]. Future RCTs with longer treatment durations (i.e., at least 1 year) are needed to confirm these findings.

## Conclusion

This NMA revealed that TENS was the only intervention to demonstrate superior efficacy in both the primary outcome (i.e., reduction in pain severity) and secondary outcomes (i.e., improvement in sleep disruption). Furthermore, all investigated non-invasive brain and nerve stimulation treatments showed comparable mortality and dropout rates to those of the control groups. This study provides valuable comparative evidence supporting the use of various brain and nerve stimulation techniques in the management of diabetic neuropathy. We believed the main findings of the current NMA could help in relieving discomfort related to diabetic neuropathy so that the overall social economic status would be improved through the ameliorating disease burden. Future well-designed RCTs with extended treatment durations are recommended to further substantiate the long-term efficacy of these interventions.

## Supplementary Information

Additional file 1.

Additional file 2.

## Data Availability

The data of the current study would be available upon reasonable request.
