# Supplementary material for: The comparative evidence of efficacy of non-invasive brain and nerve stimulation in diabetic neuropathy: a systematic review and network meta-analysis
Source: J Neuroeng Rehabil. 2025 Apr 19;22:88. doi: 10.1186/s12984-025-01614-y (PMC12008842; doi:10.1186/s12984-025-01614-y)
Supplement: Supplementary file 1 — Additional file 1. [file 12984_2025_1614_MOESM1_ESM.pptx]

## Slide 1
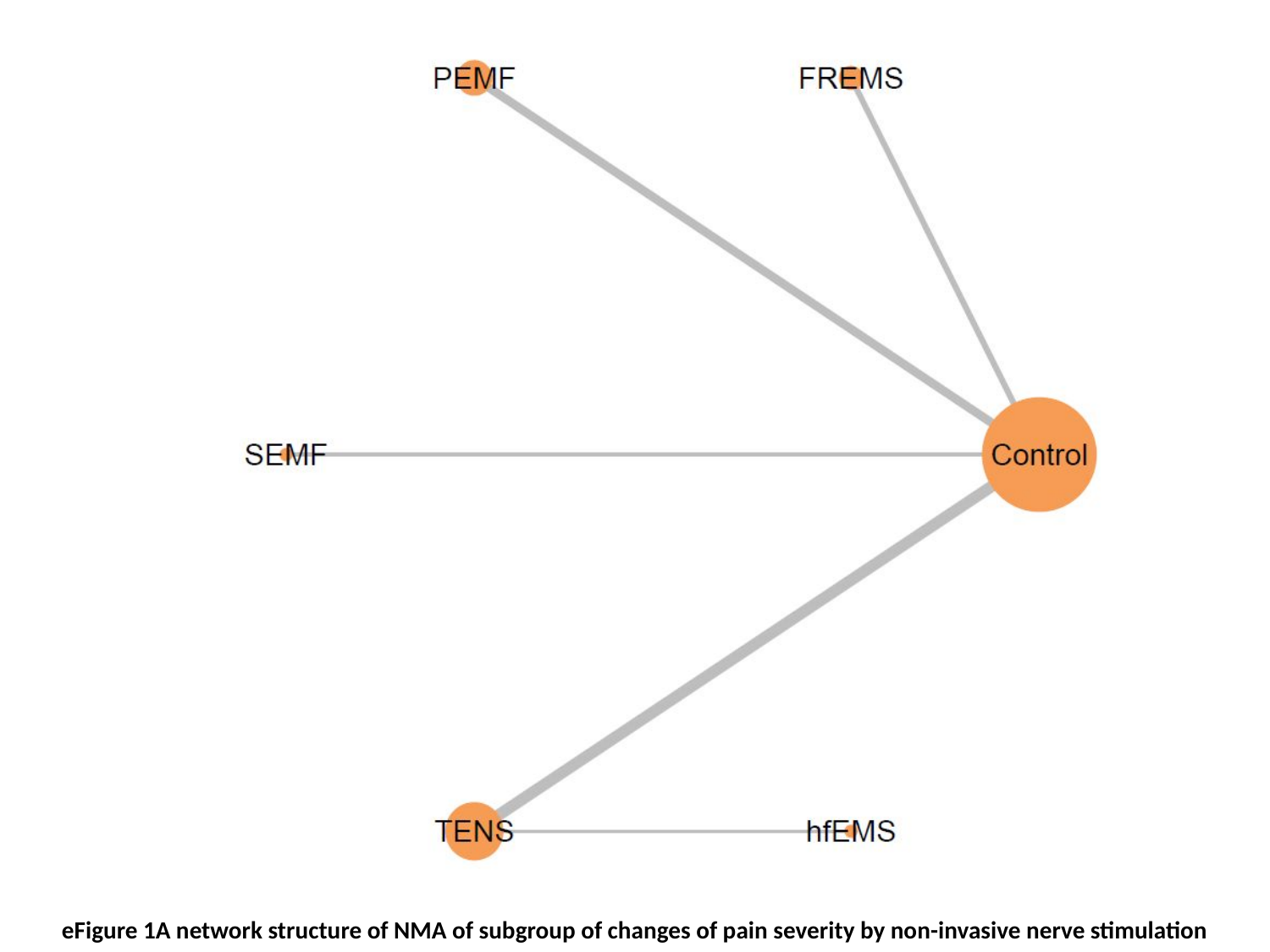

eFigure 1A network structure of NMA of subgroup of changes of pain severity by non-invasive nerve stimulation

## Slide 2
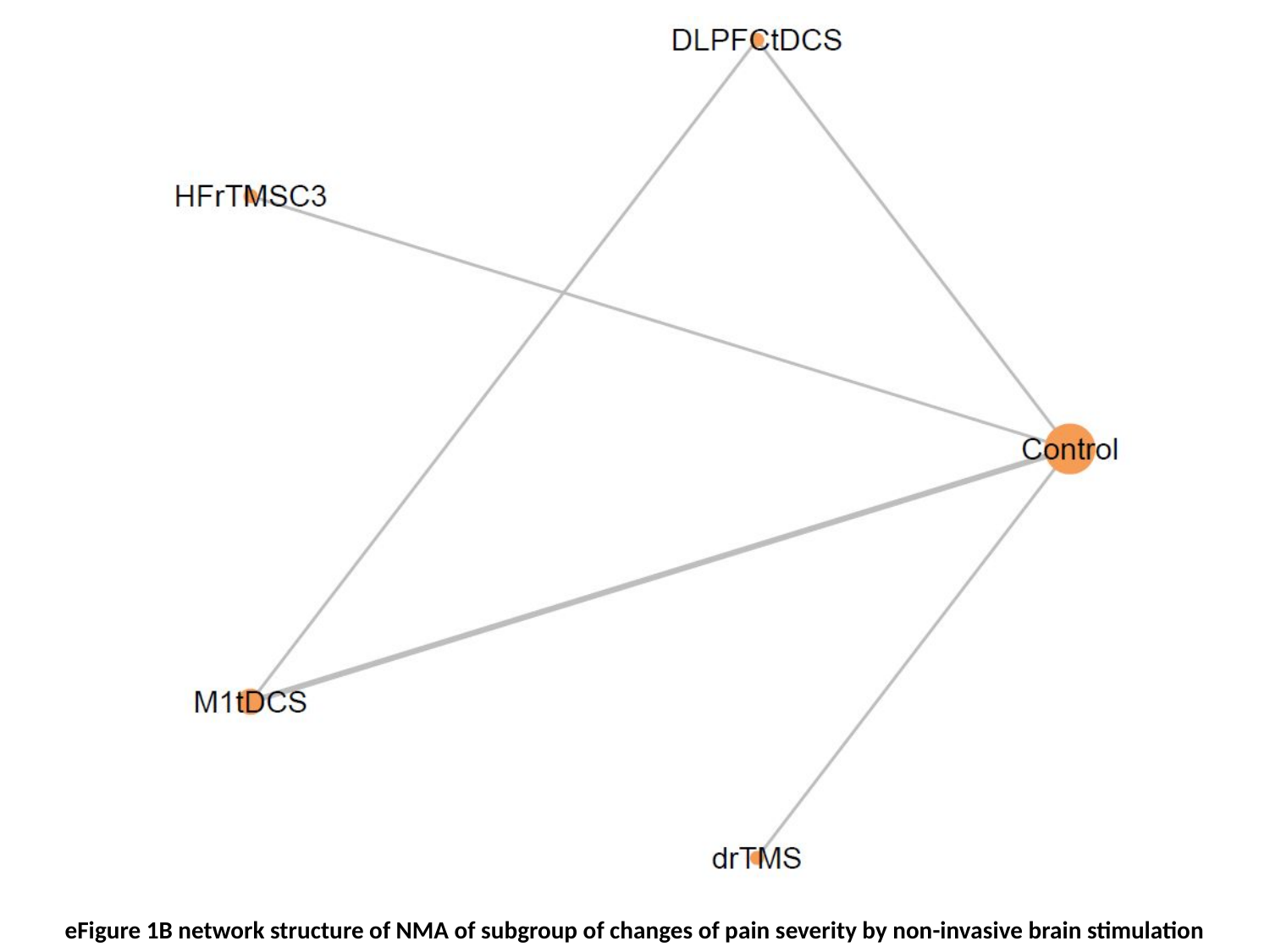

eFigure 1B network structure of NMA of subgroup of changes of pain severity by non-invasive brain stimulation

## Slide 3
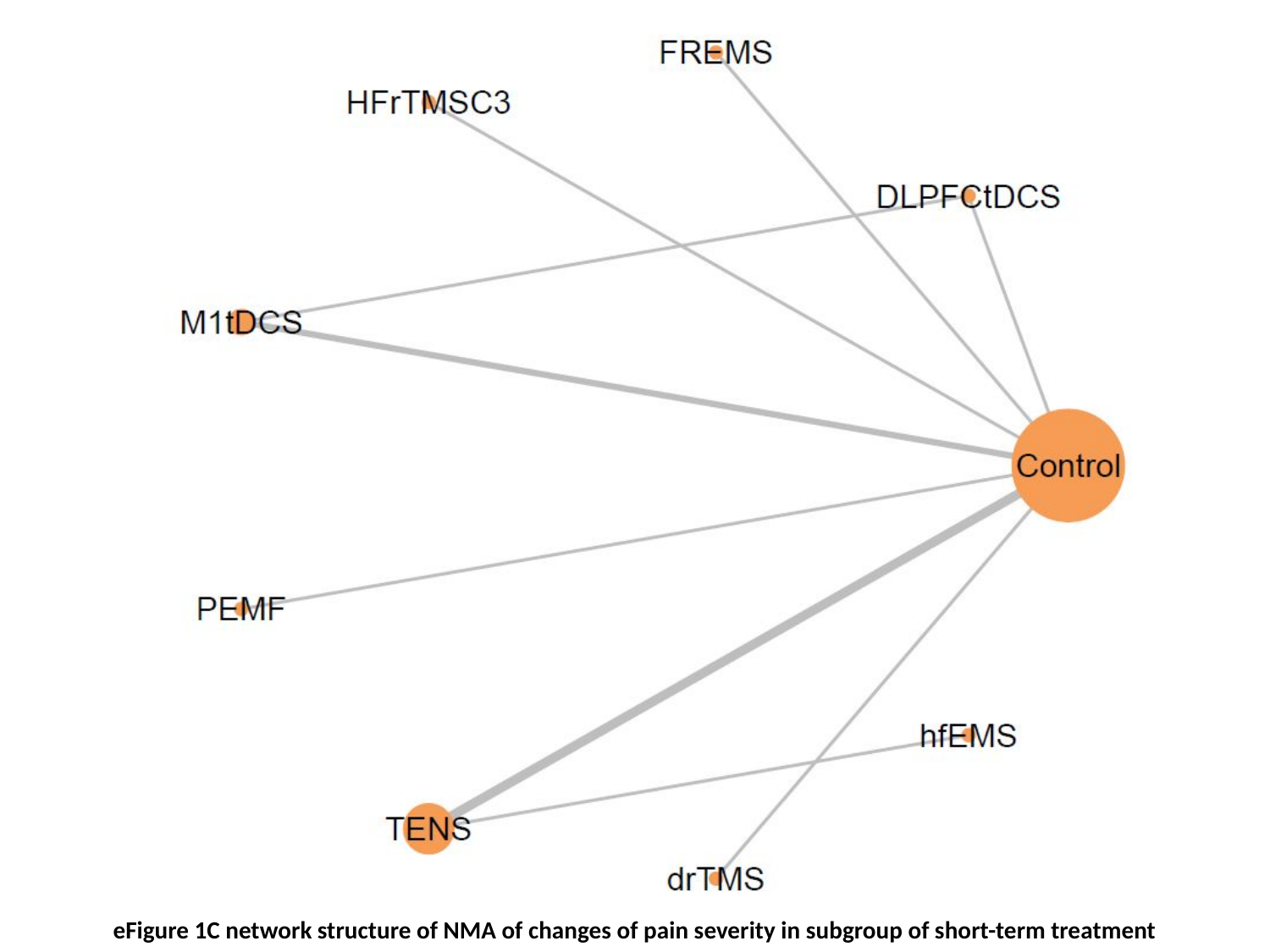

eFigure 1C network structure of NMA of changes of pain severity in subgroup of short-term treatment

## Slide 4
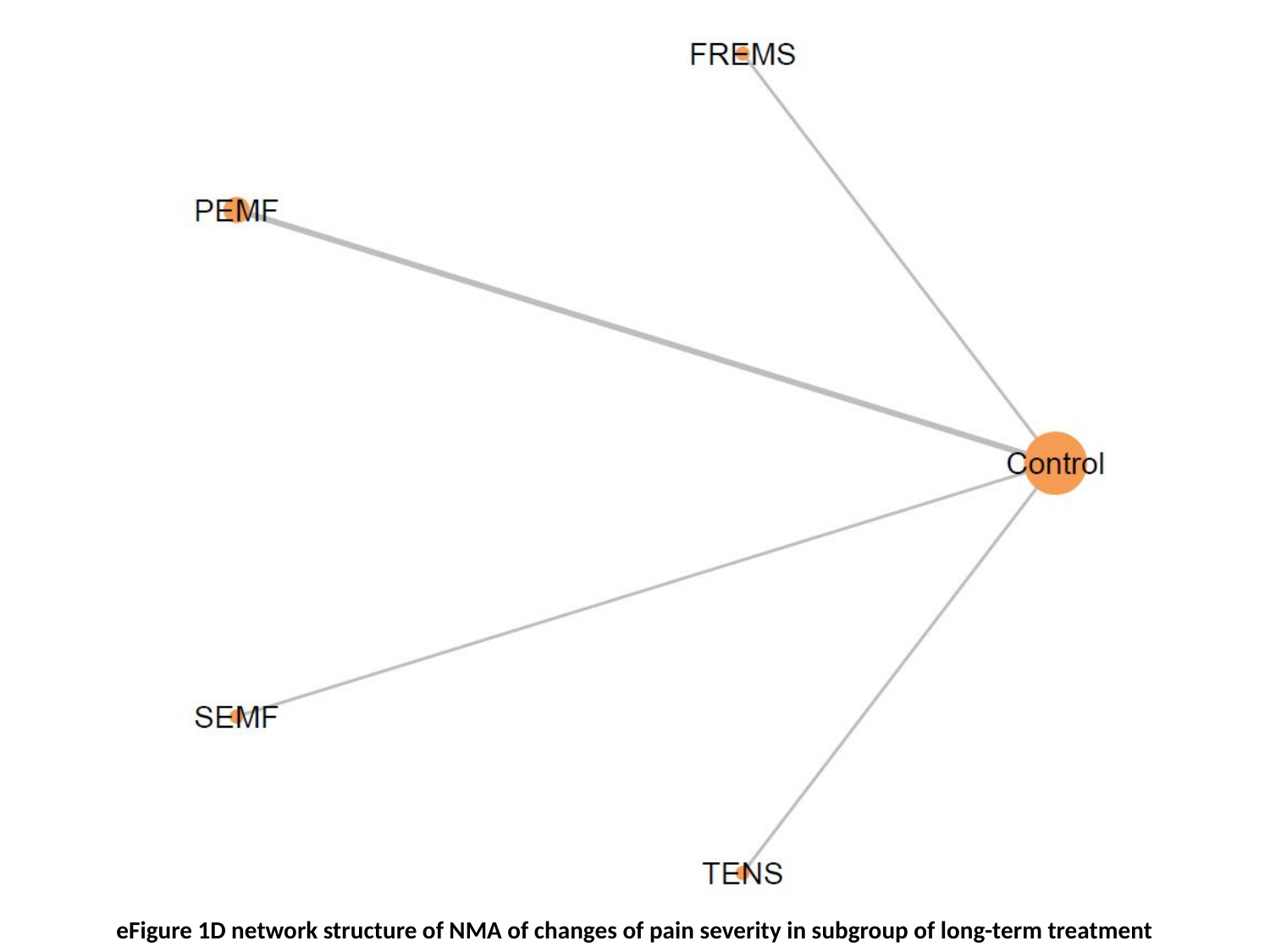

eFigure 1D network structure of NMA of changes of pain severity in subgroup of long-term treatment

## Slide 5
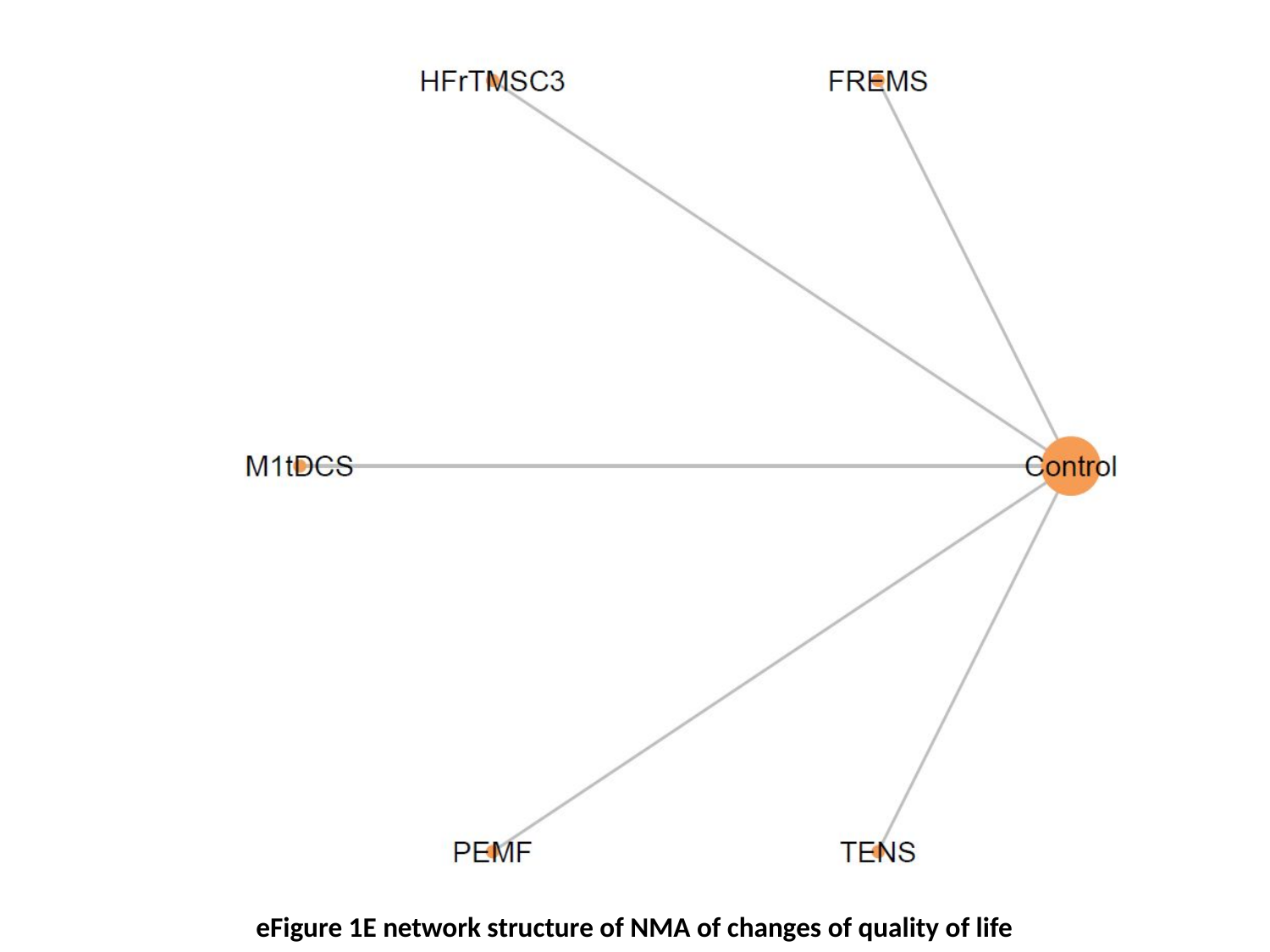

eFigure 1E network structure of NMA of changes of quality of life

## Slide 6
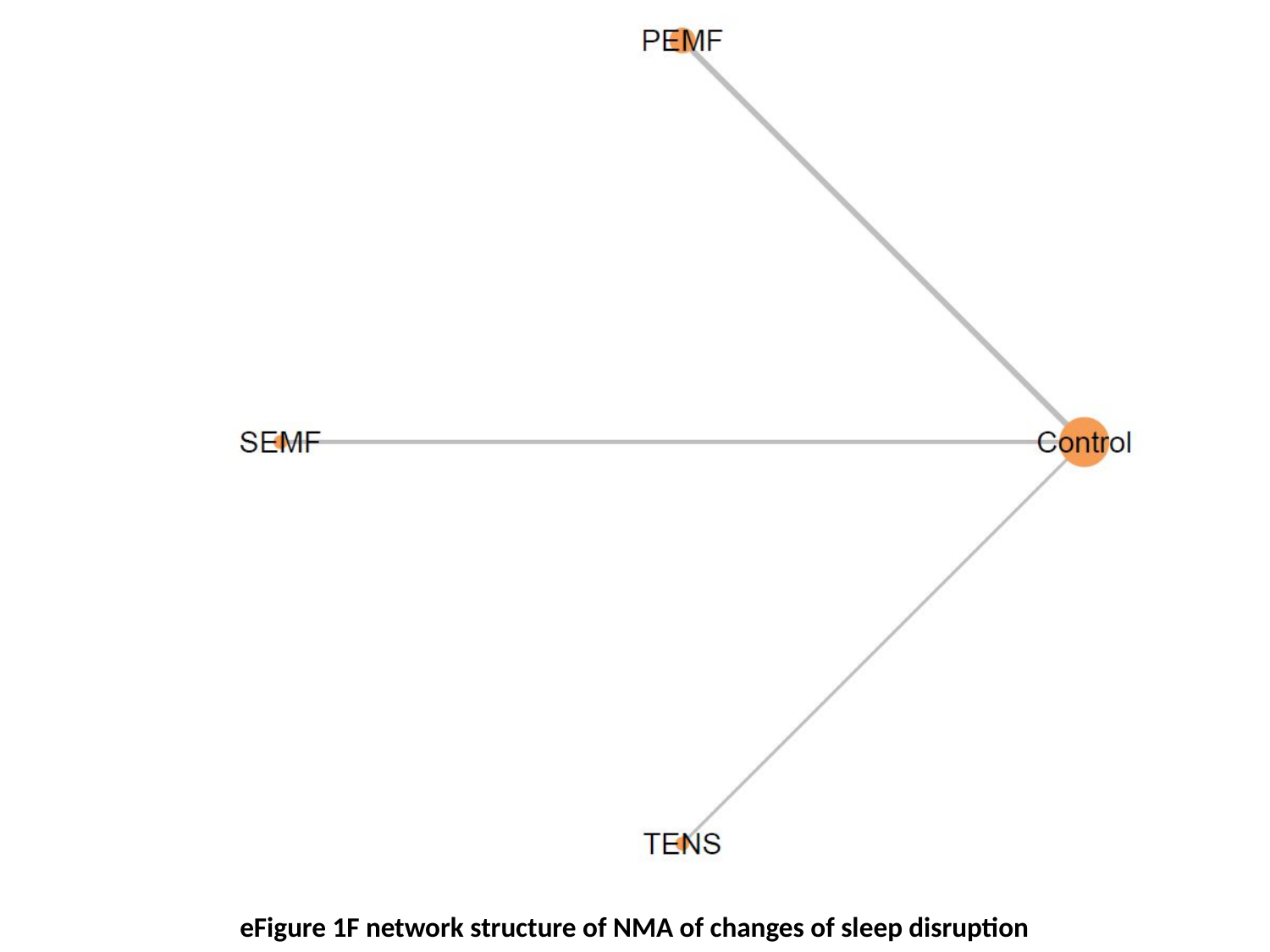

eFigure 1F network structure of NMA of changes of sleep disruption

## Slide 7
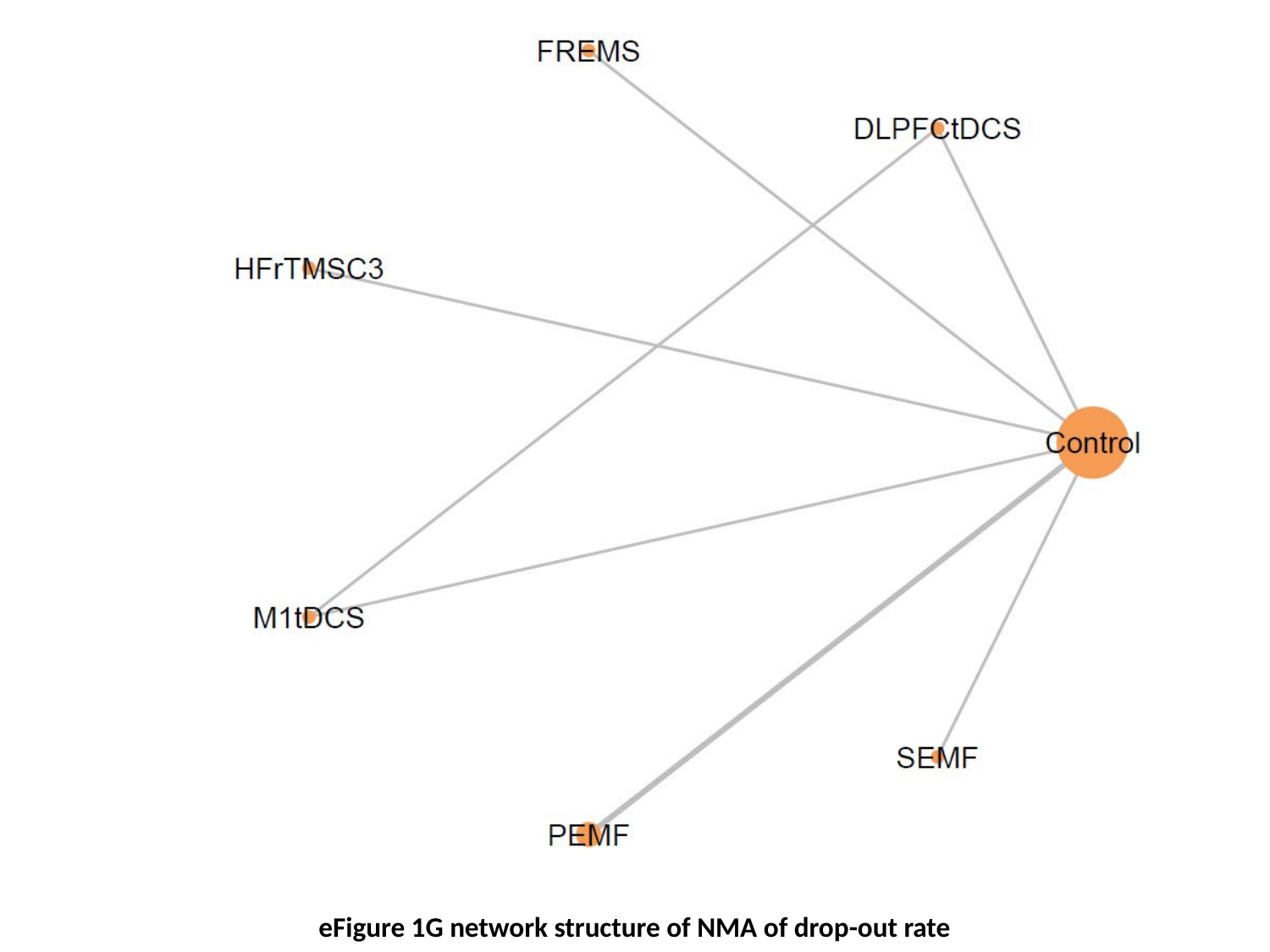

eFigure 1G network structure of NMA of drop-out rate

## Slide 8
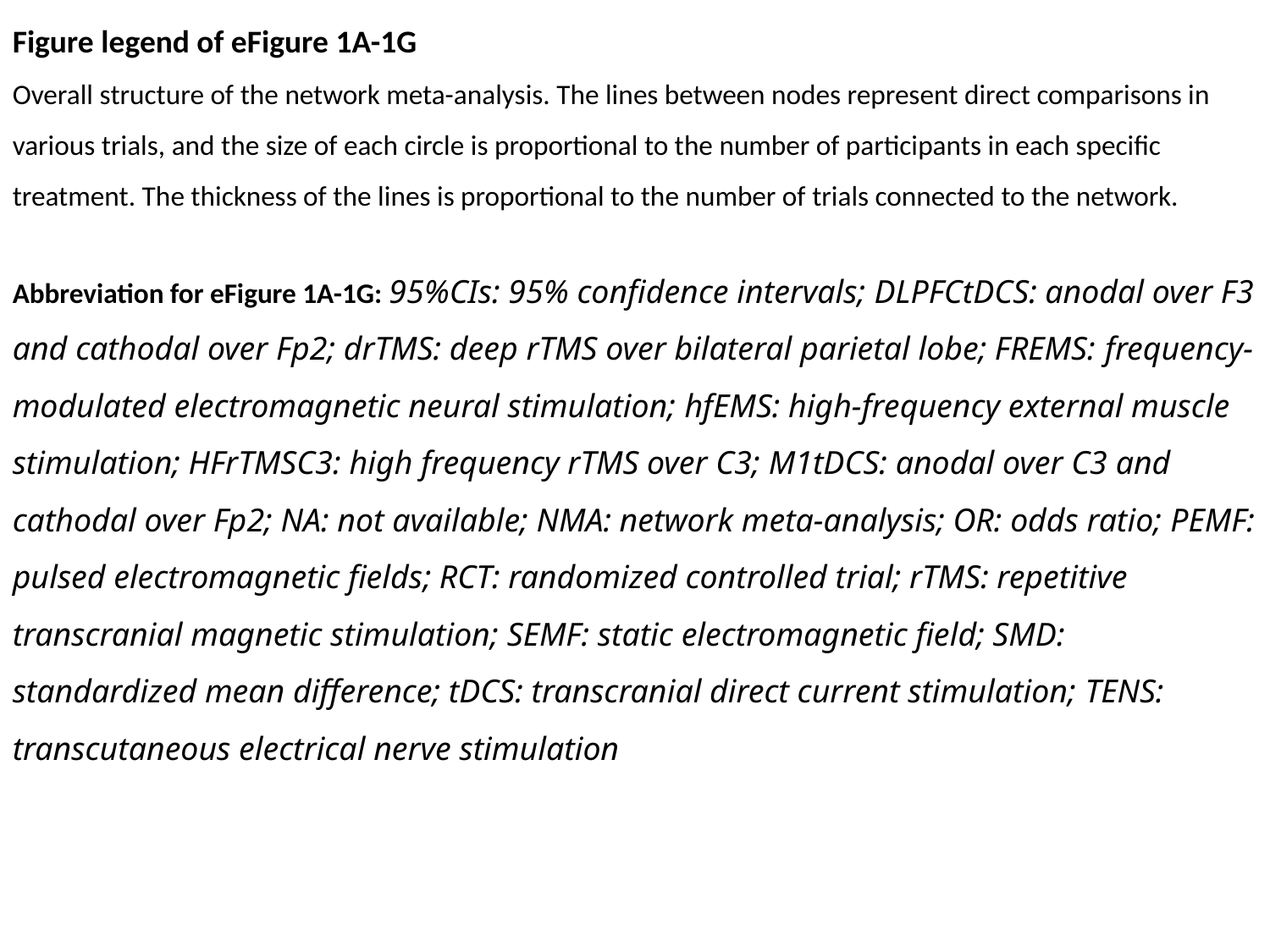

Figure legend of eFigure 1A-1G
Overall structure of the network meta-analysis. The lines between nodes represent direct comparisons in various trials, and the size of each circle is proportional to the number of participants in each specific treatment. The thickness of the lines is proportional to the number of trials connected to the network.
Abbreviation for eFigure 1A-1G: 95%CIs: 95% confidence intervals; DLPFCtDCS: anodal over F3 and cathodal over Fp2; drTMS: deep rTMS over bilateral parietal lobe; FREMS: frequency-modulated electromagnetic neural stimulation; hfEMS: high-frequency external muscle stimulation; HFrTMSC3: high frequency rTMS over C3; M1tDCS: anodal over C3 and cathodal over Fp2; NA: not available; NMA: network meta-analysis; OR: odds ratio; PEMF: pulsed electromagnetic fields; RCT: randomized controlled trial; rTMS: repetitive transcranial magnetic stimulation; SEMF: static electromagnetic field; SMD: standardized mean difference; tDCS: transcranial direct current stimulation; TENS: transcutaneous electrical nerve stimulation

## Slide 9
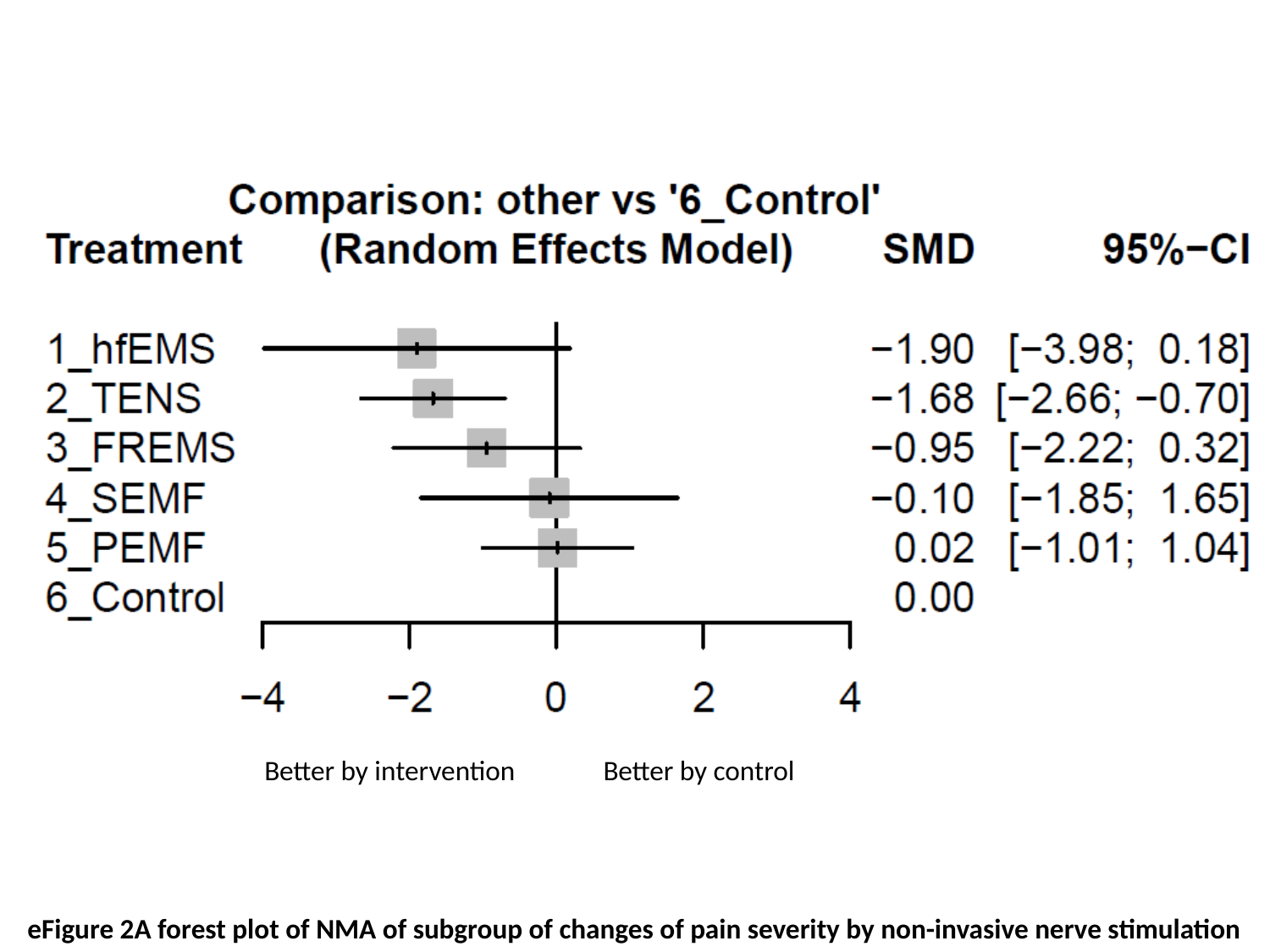

Better by intervention
Better by control
eFigure 2A forest plot of NMA of subgroup of changes of pain severity by non-invasive nerve stimulation

## Slide 10
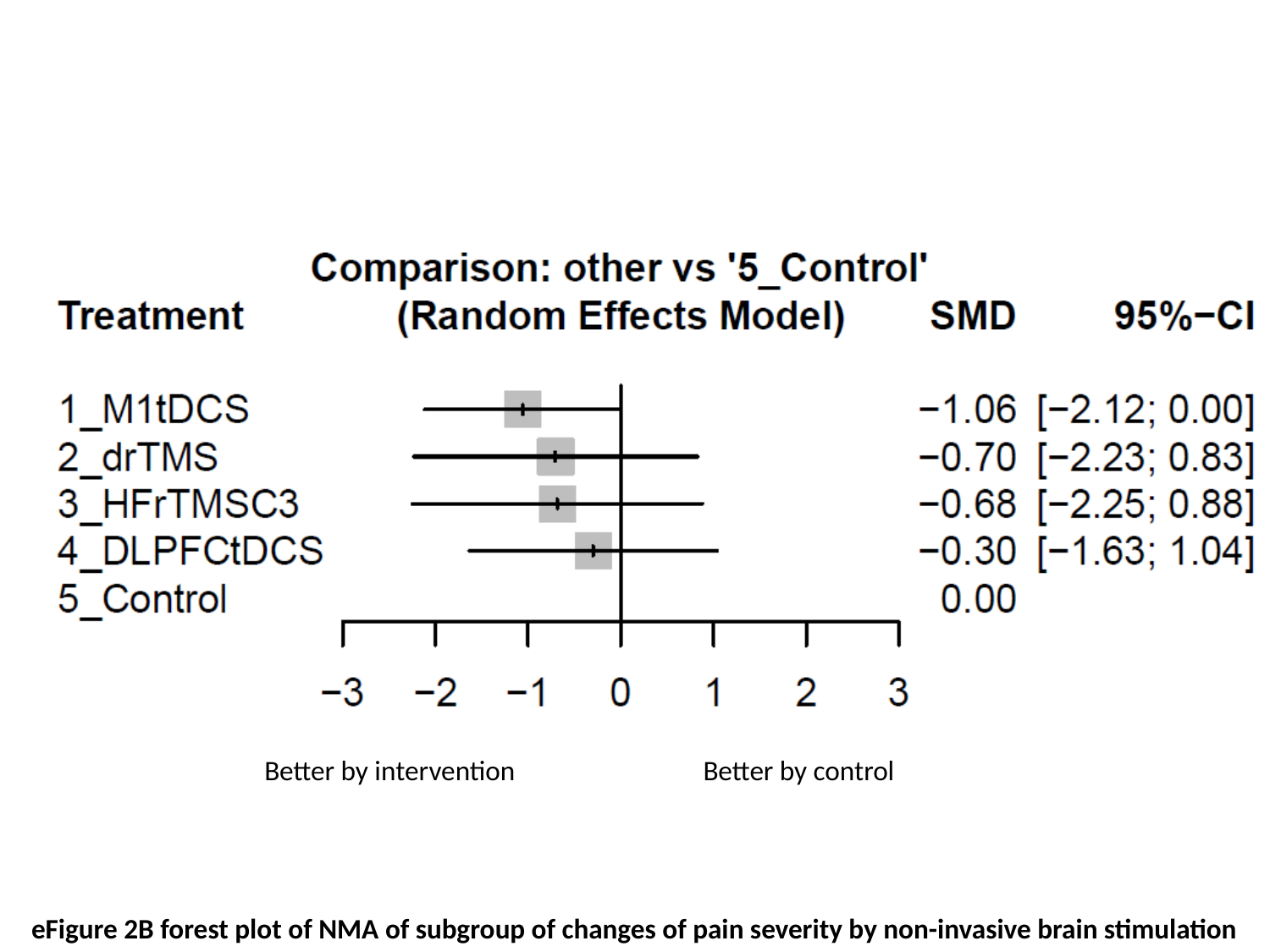

Better by intervention
Better by control
eFigure 2B forest plot of NMA of subgroup of changes of pain severity by non-invasive brain stimulation

## Slide 11
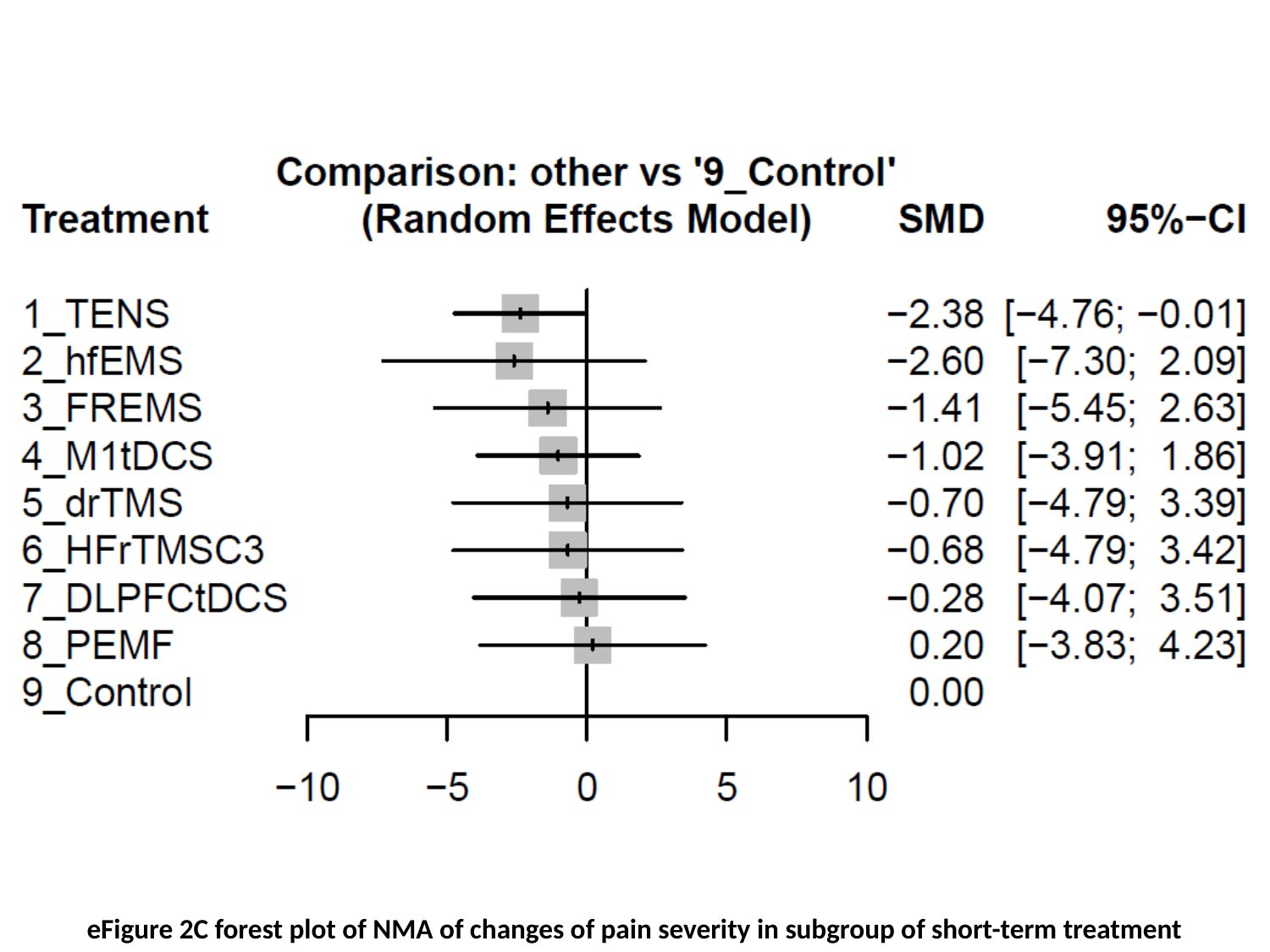

eFigure 2C forest plot of NMA of changes of pain severity in subgroup of short-term treatment

## Slide 12
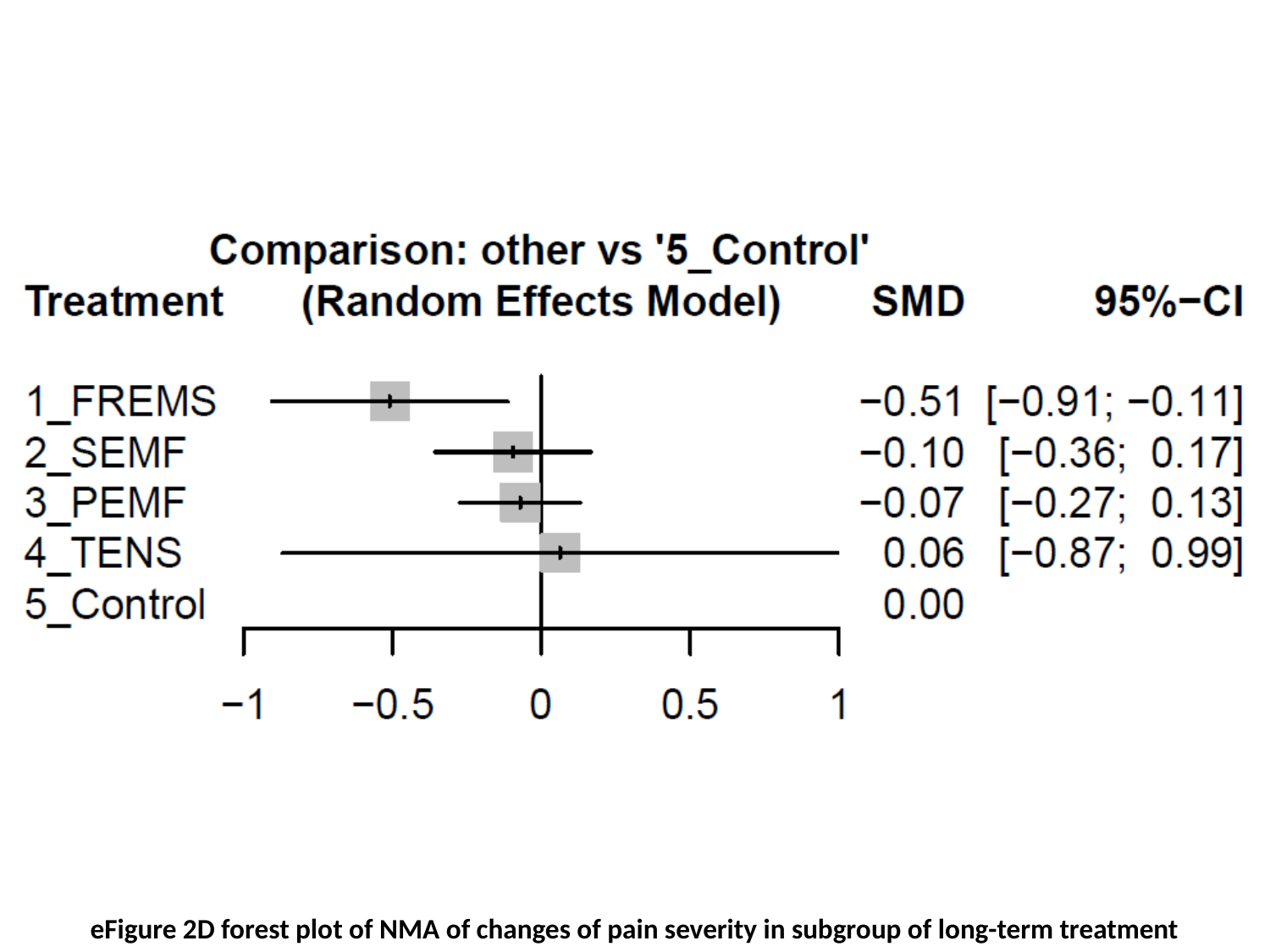

eFigure 2D forest plot of NMA of changes of pain severity in subgroup of long-term treatment

## Slide 13
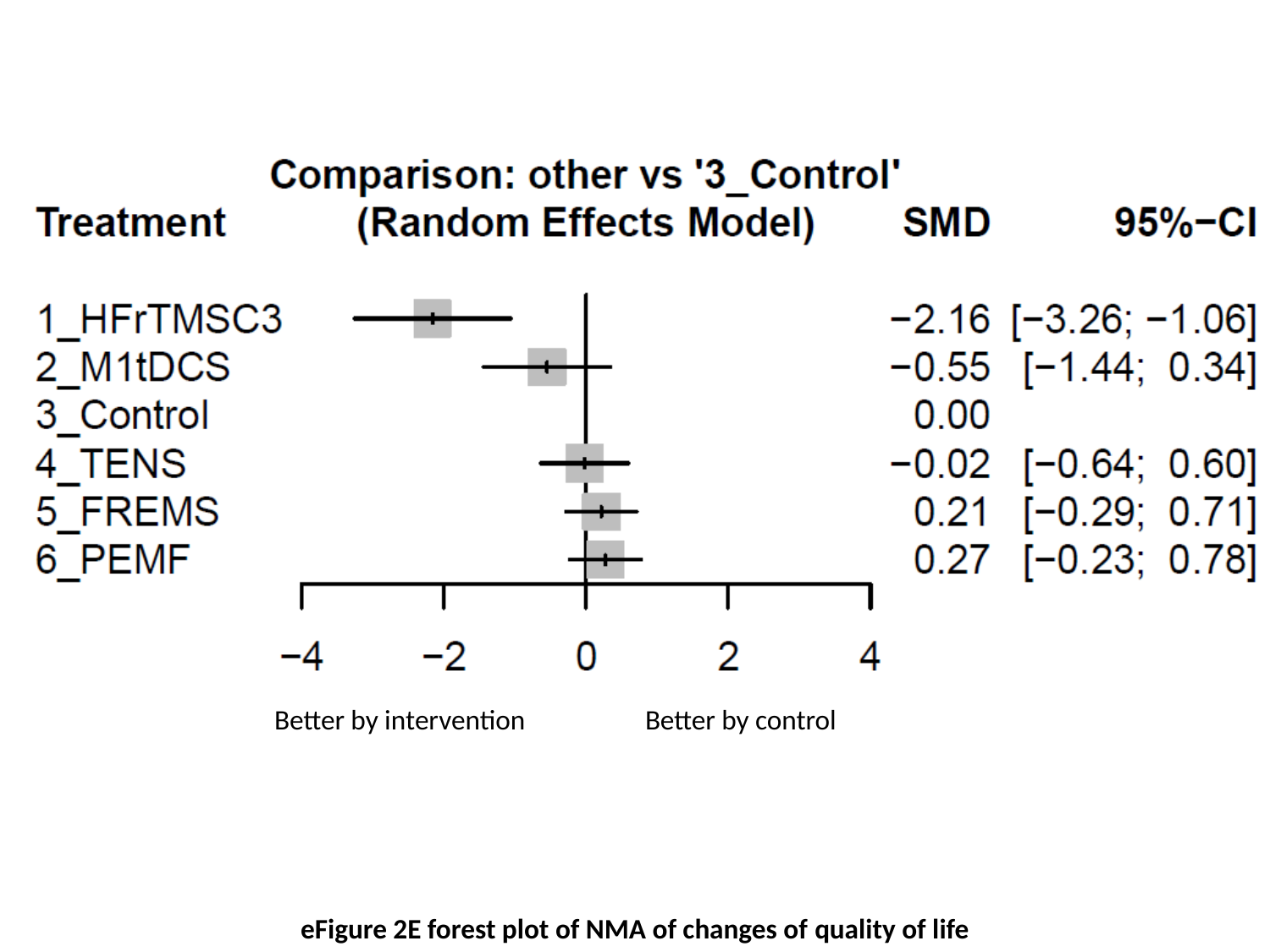

Better by intervention
Better by control
eFigure 2E forest plot of NMA of changes of quality of life

## Slide 14
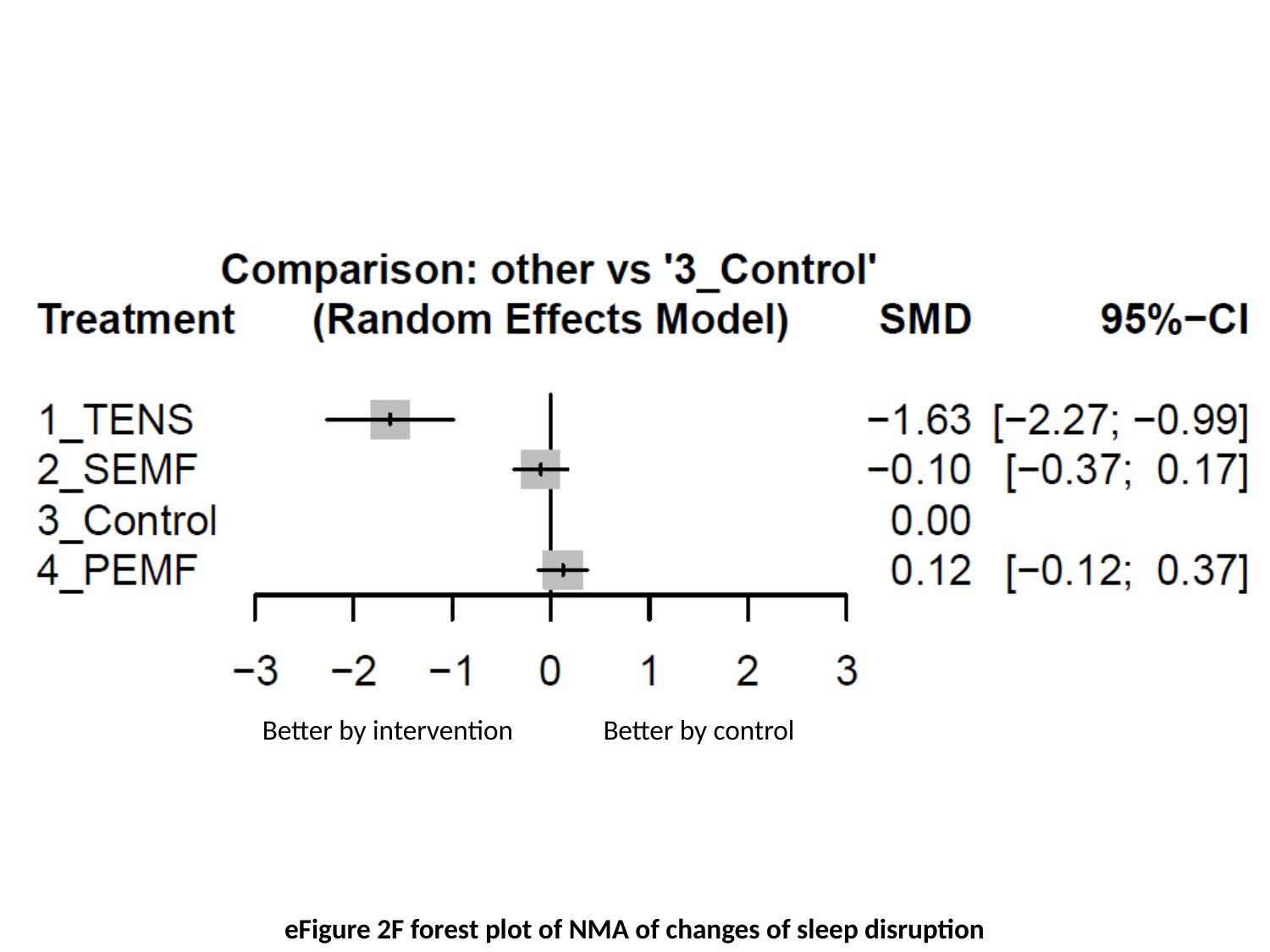

Better by intervention
Better by control
eFigure 2F forest plot of NMA of changes of sleep disruption

## Slide 15
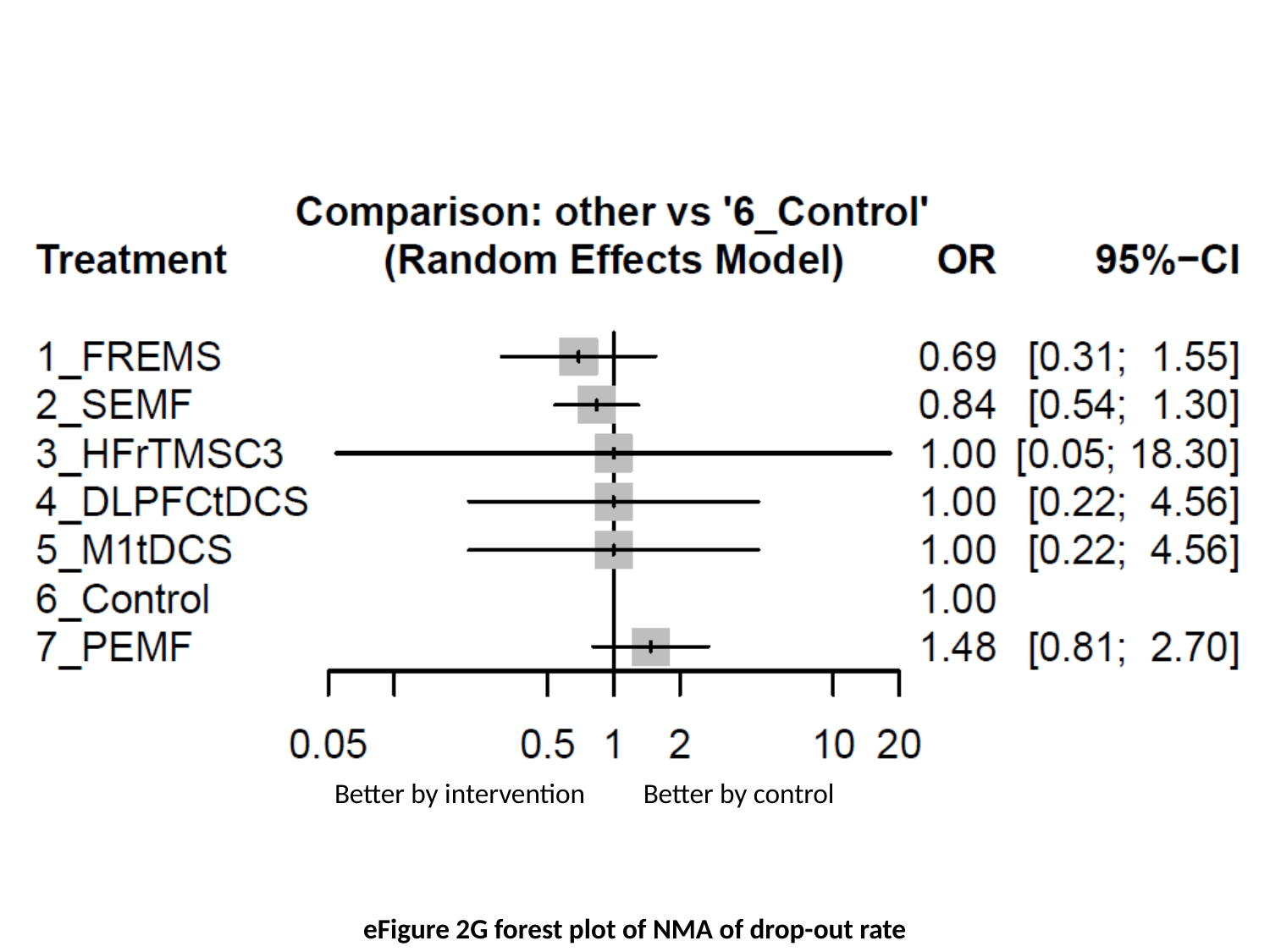

Better by intervention
Better by control
eFigure 2G forest plot of NMA of drop-out rate

## Slide 16
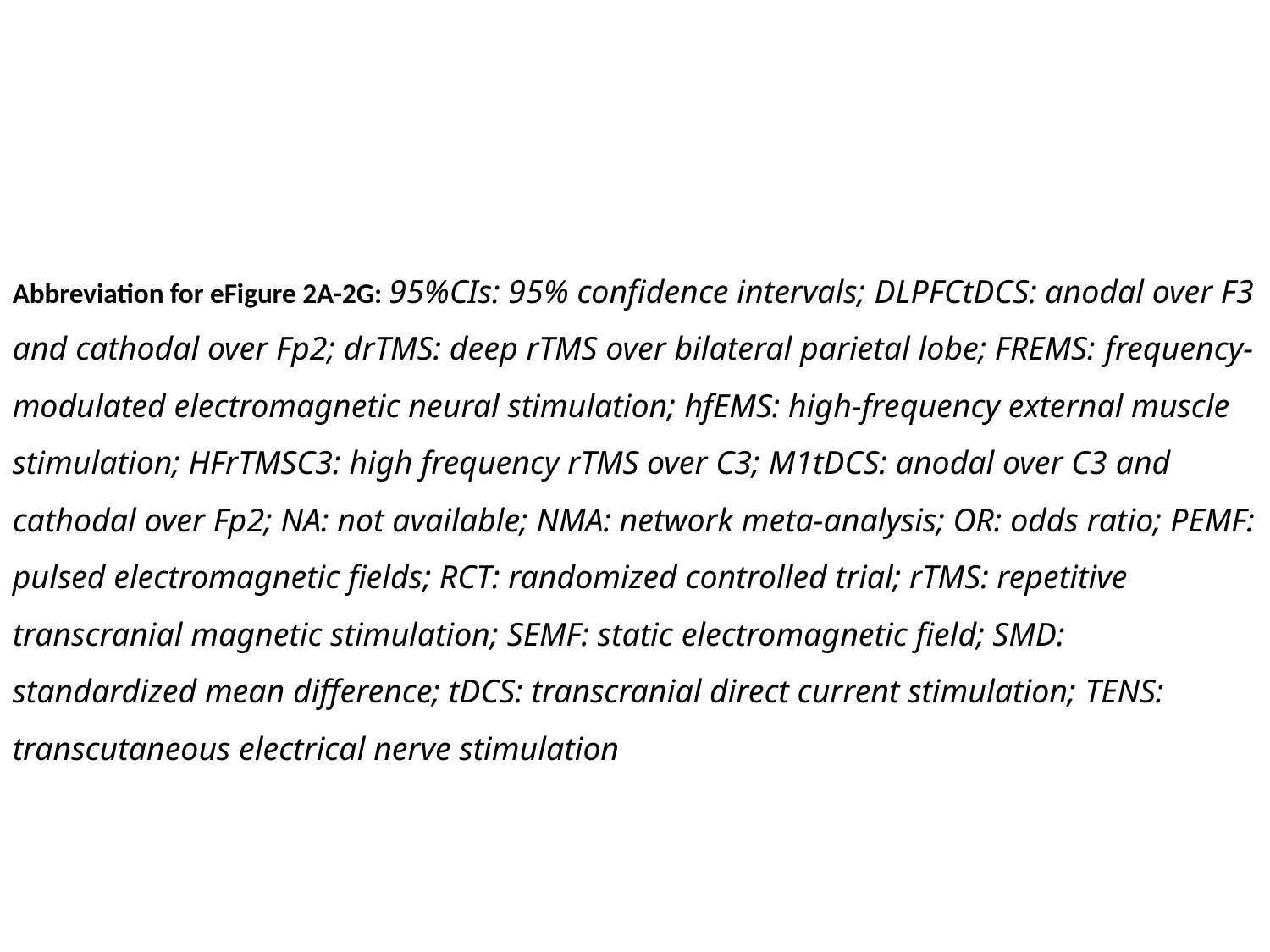

Abbreviation for eFigure 2A-2G: 95%CIs: 95% confidence intervals; DLPFCtDCS: anodal over F3 and cathodal over Fp2; drTMS: deep rTMS over bilateral parietal lobe; FREMS: frequency-modulated electromagnetic neural stimulation; hfEMS: high-frequency external muscle stimulation; HFrTMSC3: high frequency rTMS over C3; M1tDCS: anodal over C3 and cathodal over Fp2; NA: not available; NMA: network meta-analysis; OR: odds ratio; PEMF: pulsed electromagnetic fields; RCT: randomized controlled trial; rTMS: repetitive transcranial magnetic stimulation; SEMF: static electromagnetic field; SMD: standardized mean difference; tDCS: transcranial direct current stimulation; TENS: transcutaneous electrical nerve stimulation

## Slide 17
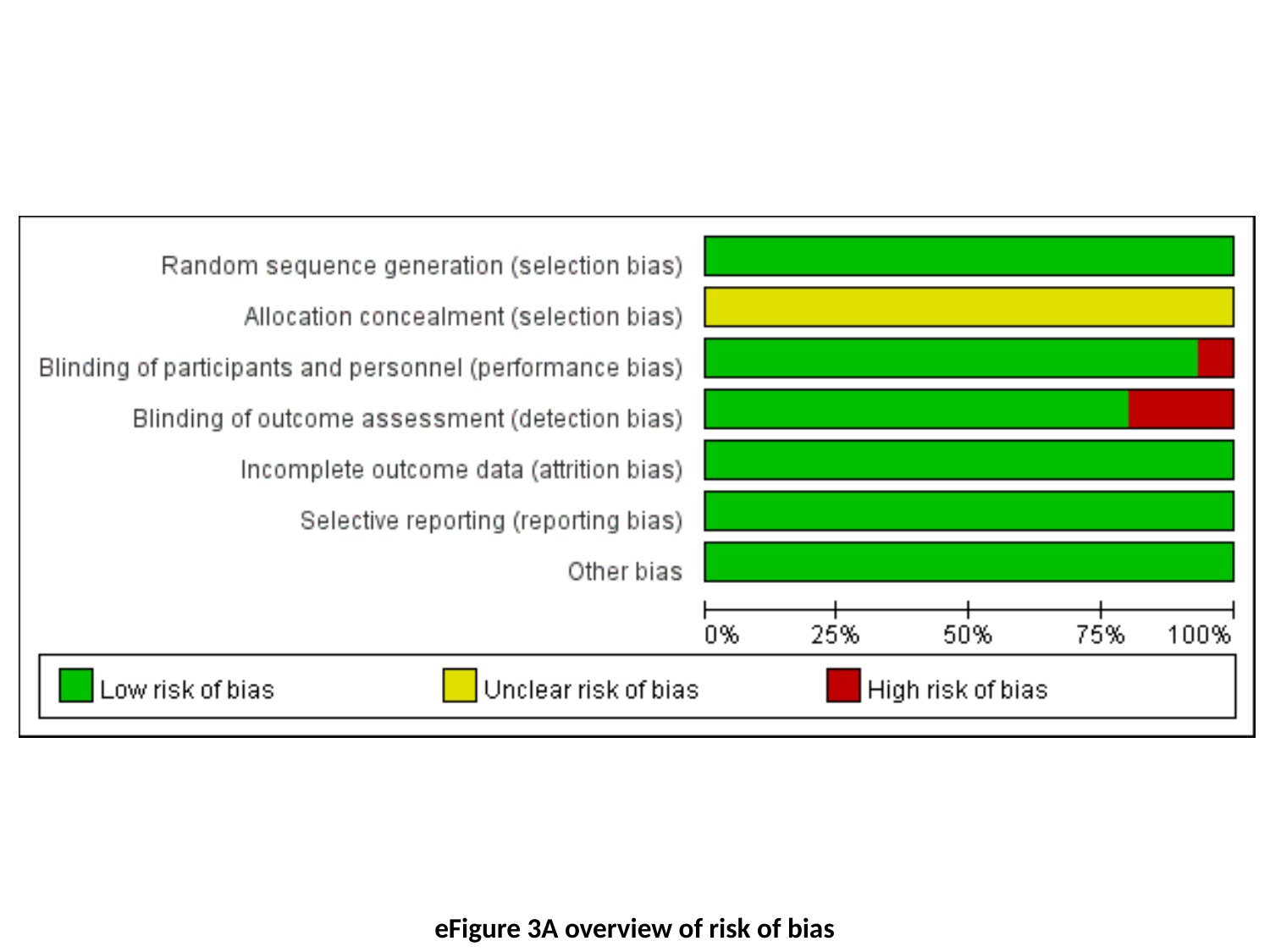

eFigure 3A overview of risk of bias

## Slide 18
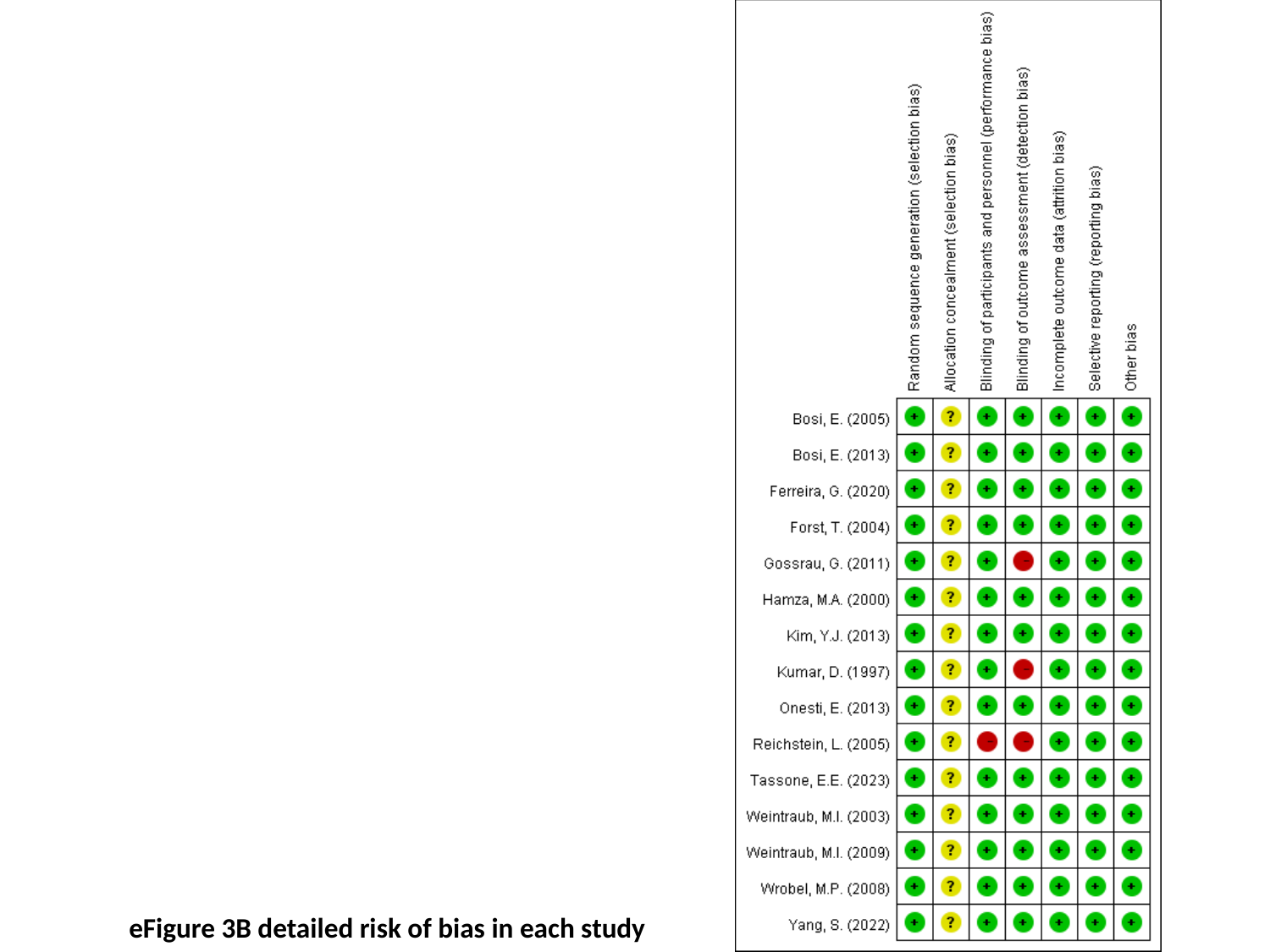

eFigure 3B detailed risk of bias in each study
