# Supplementary material for: The comparative evidence of efficacy of non-invasive brain and nerve stimulation in diabetic neuropathy: a systematic review and network meta-analysis
Source: J Neuroeng Rehabil. 2025 Apr 19;22:88. doi: 10.1186/s12984-025-01614-y (PMC12008842; doi:10.1186/s12984-025-01614-y)
Supplement: Supplementary file 2 — Additional file 2. [file 12984_2025_1614_MOESM2_ESM.docx]

**eTable 1:** PRISMA 2020 checklist of the current network meta-analysis

| **Section and Topic** | **Item #** | **Checklist item** | **Page where item is reported** |
| --- | --- | --- | --- |
| **TITLE** | | |  |
| Title | 1 | Identify the report as a systematic review. | 1 |
| **ABSTRACT** | | |  |
| Abstract | 2 | See the PRISMA 2020 for Abstracts checklist. | 5 |
| **INTRODUCTION** | | |  |
| Rationale | 3 | Describe the rationale for the review in the context of existing knowledge. | 7-8 |
| Objectives | 4 | Provide an explicit statement of the objective(s) or question(s) the review addresses. | 7-8 |
| **METHODS** | | |  |
| Eligibility criteria | 5 | Specify the inclusion and exclusion criteria for the review and how studies were grouped for the syntheses. | 9-10 |
| Information sources | 6 | Specify all databases, registers, websites, organisations, reference lists and other sources searched or consulted to identify studies. Specify the date when each source was last searched or consulted. | 9-10 |
| Search strategy | 7 | Present the full search strategies for all databases, registers and websites, including any filters and limits used. | 9-10 |
| Selection process | 8 | Specify the methods used to decide whether a study met the inclusion criteria of the review, including how many reviewers screened each record and each report retrieved, whether they worked independently, and if applicable, details of automation tools used in the process. | 9-10 |
| Data collection process | 9 | Specify the methods used to collect data from reports, including how many reviewers collected data from each report, whether they worked independently, any processes for obtaining or confirming data from study investigators, and if applicable, details of automation tools used in the process. | 9-10 |
| Data items | 10a | List and define all outcomes for which data were sought. Specify whether all results that were compatible with each outcome domain in each study were sought (e.g. for all measures, time points, analyses), and if not, the methods used to decide which results to collect. | 10-11 |
|  | 10b | List and define all other variables for which data were sought (e.g. participant and intervention characteristics, funding sources). Describe any assumptions made about any missing or unclear information. | 10-11 |
| Study risk of bias assessment | 11 | Specify the methods used to assess risk of bias in the included studies, including details of the tool(s) used, how many reviewers assessed each study and whether they worked independently, and if applicable, details of automation tools used in the process. | 10-11 |
| Effect measures | 12 | Specify for each outcome the effect measure(s) (e.g. risk ratio, mean difference) used in the synthesis or presentation of results. | 10-11 |
| Synthesis methods | 13a | Describe the processes used to decide which studies were eligible for each synthesis (e.g. tabulating the study intervention characteristics and comparing against the planned groups for each synthesis (item #5)). | 11-12 |
|  | 13b | Describe any methods required to prepare the data for presentation or synthesis, such as handling of missing summary statistics, or data conversions. | 11-12 |
|  | 13c | Describe any methods used to tabulate or visually display results of individual studies and syntheses. | 11-12 |
|  | 13d | Describe any methods used to synthesize results and provide a rationale for the choice(s). If meta-analysis was performed, describe the model(s), method(s) to identify the presence and extent of statistical heterogeneity, and software package(s) used. | 11-12 |
|  | 13e | Describe any methods used to explore possible causes of heterogeneity among study results (e.g. subgroup analysis, meta-regression). | 12-13 |
|  | 13f | Describe any sensitivity analyses conducted to assess robustness of the synthesized results. | 12-13 |
| Reporting bias assessment | 14 | Describe any methods used to assess risk of bias due to missing results in a synthesis (arising from reporting biases). | 12-13 |
| Certainty assessment | 15 | Describe any methods used to assess certainty (or confidence) in the body of evidence for an outcome. | 12-13 |
| **RESULTS** | | |  |
| Study selection | 16a | Describe the results of the search and selection process, from the number of records identified in the search to the number of studies included in the review, ideally using a flow diagram. | 14-15, Fig 1, eTab 2 |
|  | 16b | Cite studies that might appear to meet the inclusion criteria, but which were excluded, and explain why they were excluded. | 14-15, eTab 3 |
| Study characteristics | 17 | Cite each included study and present its characteristics. | 14-15, Tab 1 |
| Risk of bias in studies | 18 | Present assessments of risk of bias for each included study. | 14-15, eFig 4 |
| Results of individual studies | 19 | For all outcomes, present, for each study: (a) summary statistics for each group (where appropriate) and (b) an effect estimate and its precision (e.g. confidence/credible interval), ideally using structured tables or plots. | 14-15, Tab 2 |
| Results of syntheses | 20a | For each synthesis, briefly summarise the characteristics and risk of bias among contributing studies. | 15-16, Fig 2 |
|  | 20b | Present results of all statistical syntheses conducted. If meta-analysis was done, present for each the summary estimate and its precision (e.g. confidence/credible interval) and measures of statistical heterogeneity. If comparing groups, describe the direction of the effect. | 15-16, Fig 3 |
|  | 20c | Present results of all investigations of possible causes of heterogeneity among study results. | 15-16, eTab 6-7 |
|  | 20d | Present results of all sensitivity analyses conducted to assess the robustness of the synthesized results. | 15-16 |
| Reporting biases | 21 | Present assessments of risk of bias due to missing results (arising from reporting biases) for each synthesis assessed. | 16-17, eFig 4 |
| Certainty of evidence | 22 | Present assessments of certainty (or confidence) in the body of evidence for each outcome assessed. | 16-17 |
| **DISCUSSION** | | |  |
| Discussion | 23a | Provide a general interpretation of the results in the context of other evidence. | 18-20 |
|  | 23b | Discuss any limitations of the evidence included in the review. | 20-21 |
|  | 23c | Discuss any limitations of the review processes used. | 20-21 |
|  | 23d | Discuss implications of the results for practice, policy, and future research. | 21 |
| **OTHER INFORMATION** | | |  |
| Registration and protocol | 24a | Provide registration information for the review, including register name and registration number, or state that the review was not registered. | 5 |
|  | 24b | Indicate where the review protocol can be accessed, or state that a protocol was not prepared. | 5 |
|  | 24c | Describe and explain any amendments to information provided at registration or in the protocol. | 5 |
| Support | 25 | Describe sources of financial or non-financial support for the review, and the role of the funders or sponsors in the review. | 22 |
| Competing interests | 26 | Declare any competing interests of review authors. | 22 |
| Availability of data, code and other materials | 27 | Report which of the following are publicly available and where they can be found: template data collection forms; data extracted from included studies; data used for all analyses; analytic code; any other materials used in the review. | 22 |

The current checklist followed the latest PRISMA 2020 guideline [1].

**eTable 2: Keyword used in each database and search results**

| Database | Keyword | Filter | Date | Result |
| --- | --- | --- | --- | --- |
| PubMed | (transcutaneous electrical nerve stimulator OR TENS OR pulsed electromagnetic field OR PEMF OR deep transcranial magnetic stimulation OR dTMS OR repetitive transcranial magnetic stimulation OR rTMS OR TMS OR non-invasive brain stimulation OR non-invasive nerve stimulation OR theta burst stimulation OR transcranial direct current stimulation OR TBS OR tDCS OR vagus nerve stimulation OR vagal nerve stimulation OR tVNS OR nVNS OR VNS OR static magnetic field stimulation OR colon electric stimulation) AND (diabetic neuropathy OR diabetic polyneuropathy) AND (random OR randomized OR randomised) | N/A | 2024/9/06 | 52 |
| Embase | (transcutaneous electrical nerve stimulator OR TENS OR pulsed electromagnetic field OR PEMF OR deep transcranial magnetic stimulation OR dTMS OR repetitive transcranial magnetic stimulation OR rTMS OR TMS OR non-invasive brain stimulation OR non-invasive nerve stimulation OR theta burst stimulation OR transcranial direct current stimulation OR TBS OR tDCS OR vagus nerve stimulation OR vagal nerve stimulation OR tVNS OR nVNS OR VNS OR static magnetic field stimulation OR colon electric stimulation) AND (diabetic neuropathy OR diabetic polyneuropathy) AND (random OR randomized OR randomised) | N/A | 2024/9/06 | 84 |
| ClinicalKey | (non-invasive brain stimulation OR non-invasive nerve stimulation) AND (diabetic neuropathy) | N/A | 2024/9/06 | 1496 |
| Cochrane CENTRAL | (transcutaneous electrical nerve stimulator OR TENS OR pulsed electromagnetic field OR PEMF OR deep transcranial magnetic stimulation OR dTMS OR repetitive transcranial magnetic stimulation OR rTMS OR TMS OR non-invasive brain stimulation OR non-invasive nerve stimulation OR theta burst stimulation OR transcranial direct current stimulation OR TBS OR tDCS OR vagus nerve stimulation OR vagal nerve stimulation OR tVNS OR nVNS OR VNS OR static magnetic field stimulation OR colon electric stimulation) AND (diabetic neuropathy OR diabetic polyneuropathy) AND (random OR randomized OR randomised) | N/A | 2024/9/06 | 135 |
| ProQuest | (non-invasive brain stimulation OR non-invasive nerve stimulation) AND (diabetic neuropathy) | N/A | 2024/9/06 | 2449 |
| ScienceDirect | (non-invasive brain stimulation OR non-invasive nerve stimulation) AND (diabetic neuropathy) | N/A | 2024/9/06 | 5552 |
| Web of Science | (non-invasive brain stimulation OR non-invasive nerve stimulation) AND (diabetic neuropathy) | N/A | 2024/9/06 | 20 |
| ClinicalTrials.gov | (non-invasive brain stimulation OR non-invasive nerve stimulation) AND (diabetic neuropathy) | N/A | 2024/9/06 | 3 |

Abbreviation: N/A: not applied

**eTable 3: Excluded studies and reason**

| Reason | Numbers | References |
| --- | --- | --- |
| Animal study | 1 | [2] |
| Both treatment arms were the same treatment modalities | 1 | [3] |
| Commentary | 1 | [4] |
| Duplicate sample source with other included studies | 1 | [5] |
| End-stage renal disease but not diabetes mellitus | 1 | [6] |
| Insufficient data | 1 | [7] |
| Not randomized controlled trial | 3 | [8-10] |
| Not related to diabetic neuropathic pain | 2 | [11,12] |
| Not related to noninvasive brain or nerve stimulation | 3 | [13-15] |
| Not related to target outcome | 4 | [16-19] |
| Only once stimulation but not a whole course of treatment | 2 | [20,21] |
| Review article | 4 | [22-25] |
| Secondary selection patients after randomization procedure (only select patients with resistant to amitriptyline after randomization) | 1 | [26] |
| Study protocol but not result of a trial | 2 | [27,28] |

**eTable 4A: League table of the subgroup of changes of pain severity by non-invasive nerve stimulation**

| hfEMS | -0.22 [-2.06; 1.61] | . | . | . | . |
| --- | --- | --- | --- | --- | --- |
| -0.22 [-2.06; 1.61] | TENS | . | . | . | ***-1.68 [-2.66; -0.70]** |
| -0.95 [-3.39; 1.49] | -0.73 [-2.33; 0.87] | FREMS | . | . | -0.95 [-2.22; 0.32] |
| -1.80 [-4.52; 0.92] | -1.58 [-3.59; 0.42] | -0.85 [-3.01; 1.31] | SEMF | . | -0.10 [-1.85; 1.65] |
| -1.91 [-4.23; 0.40] | ***-1.69 [-3.11; -0.28]** | -0.96 [-2.59; 0.67] | -0.11 [-2.14; 1.92] | PEMF | 0.02 [-1.01; 1.04] |
| -1.90 [-3.98; 0.18] | ***-1.68 [-2.66; -0.70]** | -0.95 [-2.22; 0.32] | -0.10 [-1.85; 1.65] | 0.02 [-1.01; 1.04] | Control |

Data present as SMD [95%CIs]. Pairwise (upper-right portion) and network (lower-left portion) meta-analysis results are presented as estimate effect sizes for the outcome of changes of pain severity in patients with diabetic neuropathy. Interventions are reported in order of mean ranking of beneficial effect on improvement of pain severity, and outcomes are expressed as standardized mean difference (SMD) (95% confidence intervals) (95%CIs). For the pairwise meta-analyses, SMD of less than 0 indicate that the treatment specified in the row got more beneficial effect than that specified in the column. For the network meta-analysis (NMA), SMD of less than 0 indicate that the treatment specified in the column got more beneficial effect than that specified in the row. **Bold results marked with * indicate statistical significance**.

**eTable 4B: League table of the subgroup of changes of pain severity by non-invasive brain stimulation**

| M1tDCS | . | . | -1.01 [-2.44; 0.42] | -1.06 [-2.12; 0.00] |
| --- | --- | --- | --- | --- |
| -0.36 [-2.22; 1.50] | drTMS | . | . | -0.70 [-2.23; 0.83] |
| -0.37 [-2.26; 1.51] | -0.02 [-2.20; 2.17] | HFrTMSC3 | . | -0.68 [-2.25; 0.88] |
| -0.76 [-2.10; 0.58] | -0.40 [-2.44; 1.63] | -0.39 [-2.44; 1.67] | DLPFCtDCS | -0.55 [-1.97; 0.88] |
| -1.06 [-2.12; 0.00] | -0.70 [-2.23; 0.83] | -0.68 [-2.25; 0.88] | -0.30 [-1.63; 1.04] | Control |

Data present as SMD [95%CIs]. Pairwise (upper-right portion) and network (lower-left portion) meta-analysis results are presented as estimate effect sizes for the outcome of changes of pain severity in patients with diabetic neuropathy. Interventions are reported in order of mean ranking of beneficial effect on improvement of pain severity, and outcomes are expressed as standardized mean difference (SMD) (95% confidence intervals) (95%CIs). For the pairwise meta-analyses, SMD of less than 0 indicate that the treatment specified in the row got more beneficial effect than that specified in the column. For the network meta-analysis (NMA), SMD of less than 0 indicate that the treatment specified in the column got more beneficial effect than that specified in the row. **Bold results marked with * indicate statistical significance**.

**eTable 4C: League table of the changes of pain severity of subgroup of short-term treatment duration**

| TENS | 0.22 [-3.83; 4.27] | . | . | . | . | . | . | ***-2.38 [-4.76; -0.01]** |
| --- | --- | --- | --- | --- | --- | --- | --- | --- |
| 0.22 [-3.83; 4.27] | hfEMS | . | . | . | . | . | . | . |
| -0.98 [-5.66; 3.71] | -1.20 [-7.39; 4.99] | FREMS | . | . | . | . | . | -1.41 [-5.45; 2.63] |
| -1.36 [-5.09; 2.37] | -1.58 [-7.09; 3.93] | -0.38 [-5.35; 4.58] | M1tDCS | . | . | -1.01 [-5.07; 3.04] | . | -1.02 [-3.91; 1.86] |
| -1.68 [-6.41; 3.05] | -1.90 [-8.13; 4.32] | -0.70 [-6.45; 5.05] | -0.32 [-5.33; 4.68] | drTMS | . | . | . | -0.70 [-4.79; 3.39] |
| -1.70 [-6.44; 3.04] | -1.92 [-8.15; 4.31] | -0.72 [-6.48; 5.04] | -0.34 [-5.35; 4.68] | -0.02 [-5.81; 5.77] | HFrTMSC3 | . | . | -0.68 [-4.79; 3.42] |
| -2.11 [-6.58; 2.37] | -2.33 [-8.36; 3.71] | -1.13 [-6.67; 4.41] | -0.75 [-4.54; 3.05] | -0.42 [-6.00; 5.15] | -0.41 [-5.99; 5.18] | DLPFCtDCS | . | -0.55 [-4.60; 3.50] |
| -2.58 [-7.26; 2.10] | -2.80 [-8.99; 3.38] | -1.61 [-7.32; 4.10] | -1.22 [-6.18; 3.74] | -0.90 [-6.65; 4.84] | -0.88 [-6.64; 4.87] | -0.48 [-6.01; 5.06] | PEMF | 0.20 [-3.83; 4.23] |
| ***-2.38 [-4.76; -0.01]** | -2.60 [-7.30; 2.09] | -1.41 [-5.45; 2.63] | -1.02 [-3.91; 1.86] | -0.70 [-4.79; 3.39] | -0.68 [-4.79; 3.42] | -0.28 [-4.07; 3.51] | 0.20 [-3.83; 4.23] | Control |

Data present as SMD [95%CIs]. Pairwise (upper-right portion) and network (lower-left portion) meta-analysis results are presented as estimate effect sizes for the outcome of changes of pain severity in patients with diabetic neuropathy. Interventions are reported in order of mean ranking of beneficial effect on improvement of pain severity, and outcomes are expressed as standardized mean difference (SMD) (95% confidence intervals) (95%CIs). For the pairwise meta-analyses, SMD of less than 0 indicate that the treatment specified in the row got more beneficial effect than that specified in the column. For the network meta-analysis (NMA), SMD of less than 0 indicate that the treatment specified in the column got more beneficial effect than that specified in the row. **Bold results marked with * indicate statistical significance**.

**eTable 4D: League table of the changes of pain severity of subgroup of long-term treatment duration**

| FREMS | . | . | . | ***-0.51 [-0.91; -0.11]** |
| --- | --- | --- | --- | --- |
| -0.41 [-0.89; 0.06] | SEMF | . | . | -0.10 [-0.36; 0.17] |
| -0.44 [-0.88; 0.01] | -0.02 [-0.35; 0.31] | PEMF | . | -0.07 [-0.27; 0.13] |
| -0.57 [-1.59; 0.44] | -0.16 [-1.13; 0.81] | -0.13 [-1.09; 0.82] | TENS | 0.06 [-0.87; 0.99] |
| ***-0.51 [-0.91; -0.11]** | -0.10 [-0.36; 0.17] | -0.07 [-0.27; 0.13] | 0.06 [-0.87; 0.99] | Control |

Data present as SMD [95%CIs]. Pairwise (upper-right portion) and network (lower-left portion) meta-analysis results are presented as estimate effect sizes for the outcome of changes of pain severity in patients with diabetic neuropathy. Interventions are reported in order of mean ranking of beneficial effect on improvement of pain severity, and outcomes are expressed as standardized mean difference (SMD) (95% confidence intervals) (95%CIs). For the pairwise meta-analyses, SMD of less than 0 indicate that the treatment specified in the row got more beneficial effect than that specified in the column. For the network meta-analysis (NMA), SMD of less than 0 indicate that the treatment specified in the column got more beneficial effect than that specified in the row. **Bold results marked with * indicate statistical significance**.

**eTable 4E: League table of the secondary outcome: changes of quality of life**

| HFrTMSC3 | . | ***-2.16 [-3.26; -1.06]** | **.** | . | . |
| --- | --- | --- | --- | --- | --- |
| ***-1.61 [-3.03; -0.19]** | M1tDCS | -0.55 [-1.44; 0.34] | . | . | . |
| ***-2.16 [-3.26; -1.06]** | -0.55 [-1.44; 0.34] | Control | 0.02 [-0.60; 0.64] | -0.21 [-0.71; 0.29] | -0.27 [-0.78; 0.23] |
| ***-2.14 [-3.41; -0.87]** | -0.53 [-1.62; 0.56] | 0.02 [-0.60; 0.64] | TENS | . | . |
| ***-2.37 [-3.58; -1.16]** | -0.76 [-1.79; 0.26] | -0.21 [-0.71; 0.29] | -0.23 [-1.03; 0.56] | FREMS | . |
| ***-2.43 [-3.64; -1.22]** | -0.82 [-1.85; 0.21] | -0.27 [-0.78; 0.23] | -0.29 [-1.09; 0.51] | -0.06 [-0.77; 0.65] | PEMF |

Data present as SMD [95%CIs]. Pairwise (upper-right portion) and network (lower-left portion) meta-analysis results are presented as estimate effect sizes for the outcome of changes of quality of life in patients with diabetic neuropathy. Interventions are reported in order of mean ranking of beneficial effect on improvement of quality of life, and outcomes are expressed as standardized mean difference (SMD) (95% confidence intervals) (95%CIs). For the pairwise meta-analyses, SMD of less than 0 indicate that the treatment specified in the row got more beneficial effect than that specified in the column. For the network meta-analysis (NMA), SMD of less than 0 indicate that the treatment specified in the column got more beneficial effect than that specified in the row. **Bold results marked with * indicate statistical significance**.

**eTable 4F: League table of the secondary outcome: changes of sleep disruption**

| TENS | . | ***-1.63 [-2.27; -0.99]** | . |
| --- | --- | --- | --- |
| ***-1.53 [-2.22; -0.83]** | SEMF | -0.10 [-0.37; 0.17] | . |
| ***-1.63 [-2.27; -0.99]** | -0.10 [-0.37; 0.17] | Control | -0.12 [-0.37; 0.12] |
| ***-1.75 [-2.44; -1.07]** | -0.22 [-0.59; 0.14] | -0.12 [-0.37; 0.12] | PEMF |

Data present as SMD [95%CIs]. Pairwise (upper-right portion) and network (lower-left portion) meta-analysis results are presented as estimate effect sizes for the outcome of changes of sleep disruption in patients with diabetic neuropathy. Interventions are reported in order of mean ranking of beneficial effect on improvement of sleep disruption, and outcomes are expressed as standardized mean difference (SMD) (95% confidence intervals) (95%CIs). For the pairwise meta-analyses, SMD of less than 0 indicate that the treatment specified in the row got more beneficial effect than that specified in the column. For the network meta-analysis (NMA), SMD of less than 0 indicate that the treatment specified in the column got more beneficial effect than that specified in the row. **Bold results marked with * indicate statistical significance**.

**eTable 4G: League table of the acceptability: drop-out rate**

| FREMS | . | . | . | . | 0.69 [0.31; 1.55] | . |
| --- | --- | --- | --- | --- | --- | --- |
| 0.83 [0.33; 2.07] | SEMF | . | . | . | 0.84 [0.54; 1.30] | . |
| 0.69 [0.03; 14.15] | 0.84 [0.04; 15.83] | HFrTMSC3 | . | . | 1.00 [0.05; 18.30] | . |
| 0.69 [0.12; 3.87] | 0.84 [0.17; 4.06] | 1.00 [0.04; 26.57] | DLPFCtDCS | 1.00 [0.22; 4.56] | 1.00 [0.22; 4.56] | . |
| 0.69 [0.12; 3.87] | 0.84 [0.17; 4.06] | 1.00 [0.04; 26.57] | 1.00 [0.22; 4.56] | M1tDCS | 1.00 [0.22; 4.56] | . |
| 0.69 [0.31; 1.55] | 0.84 [0.54; 1.30] | 1.00 [0.05; 18.30] | 1.00 [0.22; 4.56] | 1.00 [0.22; 4.56] | Control | 0.68 [0.37; 1.24] |
| 0.47 [0.17; 1.29] | 0.57 [0.27; 1.20] | 0.68 [0.03; 13.20] | 0.68 [0.13; 3.47] | 0.68 [0.13; 3.47] | 0.68 [0.37; 1.24] | PEMF |

Data present as OR [95%CIs]. Pairwise (upper-right portion) and network (lower-left portion) meta-analysis results are presented as estimate effect sizes for the outcome of drop-out rate in patients with diabetic neuropathy. Interventions are reported in order of mean ranking of preferred acceptability, and outcomes are expressed as odds ratio (OR) (95% confidence intervals) (95%CIs). For the pairwise meta-analyses, OR of less than 1 indicate that the treatment specified in the row got more preferred acceptability than that specified in the column. For the network meta-analysis (NMA), OR of less than 1 indicate that the treatment specified in the column got more preferred acceptability than that specified in the row. **Bold results marked with * indicate statistical significance**.

*Abbreviation: 95%CIs: 95% confidence intervals; DLPFCtDCS: anodal over F3 and cathodal over Fp2; drTMS: deep rTMS over bilateral parietal lobe; FREMS: frequency-modulated electromagnetic neural stimulation; hfEMS: high-frequency external muscle stimulation; HFrTMSC3: high frequency rTMS over C3; M1tDCS: anodal over C3 and cathodal over Fp2; NA: not available; NMA: network meta-analysis; OR: odds ratio; PEMF: pulsed electromagnetic fields; RCT: randomized controlled trial; rTMS: repetitive transcranial magnetic stimulation; SEMF: static electromagnetic field; SMD: standardized mean difference; tDCS: transcranial direct current stimulation; TENS: transcutaneous electrical nerve stimulation*

**eTable 5: inconsistency within the network meta-analysis of primary outcome: changes of pain severity**

| Comparison | No.Studies | NMA | Direct | Indirect | Difference | Diff_95CI_lower | Diff_95CI_upper | pValue |
| --- | --- | --- | --- | --- | --- | --- | --- | --- |
| DLPFCtDCS:Control | 1 | -0.28958 | -0.54608 | 1.591506 | -2.13758 | -7.38856 | 3.113395 | 0.424947 |
| drTMS:Control | 1 | -0.70259 | -0.70259 | NA | NA | NA | NA | NA |
| FREMS:Control | 2 | -0.9471 | -0.9471 | NA | NA | NA | NA | NA |
| hfEMS:Control | 0 | -1.89655 | NA | -1.89655 | NA | NA | NA | NA |
| HFrTMSC3:Control | 1 | -0.68426 | -0.68426 | NA | NA | NA | NA | NA |
| M1tDCS:Control | 2 | -1.04364 | -1.04364 | NA | NA | NA | NA | NA |
| PEMF:Control | 3 | 0.015074 | 0.015074 | NA | NA | NA | NA | NA |
| SEMF:Control | 1 | -0.09537 | -0.09537 | NA | NA | NA | NA | NA |
| TENS:Control | 4 | -1.67499 | -1.67499 | NA | NA | NA | NA | NA |
| DLPFCtDCS:drTMS | 0 | 0.413012 | NA | 0.413012 | NA | NA | NA | NA |
| DLPFCtDCS:FREMS | 0 | 0.657524 | NA | 0.657524 | NA | NA | NA | NA |
| DLPFCtDCS:hfEMS | 0 | 1.606975 | NA | 1.606975 | NA | NA | NA | NA |
| DLPFCtDCS:HFrTMSC3 | 0 | 0.39468 | NA | 0.39468 | NA | NA | NA | NA |
| DLPFCtDCS:M1tDCS | 1 | 0.754066 | 1.014141 | -1.10894 | 2.123085 | -3.09228 | 7.338451 | 0.424947 |
| DLPFCtDCS:PEMF | 0 | -0.30465 | NA | -0.30465 | NA | NA | NA | NA |
| DLPFCtDCS:SEMF | 0 | -0.19421 | NA | -0.19421 | NA | NA | NA | NA |
| DLPFCtDCS:TENS | 0 | 1.385409 | NA | 1.385409 | NA | NA | NA | NA |
| drTMS:FREMS | 0 | 0.244512 | NA | 0.244512 | NA | NA | NA | NA |
| drTMS:hfEMS | 0 | 1.193963 | NA | 1.193963 | NA | NA | NA | NA |
| drTMS:HFrTMSC3 | 0 | -0.01833 | NA | -0.01833 | NA | NA | NA | NA |
| drTMS:M1tDCS | 0 | 0.341054 | NA | 0.341054 | NA | NA | NA | NA |
| drTMS:PEMF | 0 | -0.71766 | NA | -0.71766 | NA | NA | NA | NA |
| drTMS:SEMF | 0 | -0.60722 | NA | -0.60722 | NA | NA | NA | NA |
| drTMS:TENS | 0 | 0.972397 | NA | 0.972397 | NA | NA | NA | NA |
| FREMS:hfEMS | 0 | 0.949451 | NA | 0.949451 | NA | NA | NA | NA |
| FREMS:HFrTMSC3 | 0 | -0.26284 | NA | -0.26284 | NA | NA | NA | NA |
| FREMS:M1tDCS | 0 | 0.096542 | NA | 0.096542 | NA | NA | NA | NA |
| FREMS:PEMF | 0 | -0.96218 | NA | -0.96218 | NA | NA | NA | NA |
| FREMS:SEMF | 0 | -0.85173 | NA | -0.85173 | NA | NA | NA | NA |
| FREMS:TENS | 0 | 0.727885 | NA | 0.727885 | NA | NA | NA | NA |
| hfEMS:HFrTMSC3 | 0 | -1.2123 | NA | -1.2123 | NA | NA | NA | NA |
| hfEMS:M1tDCS | 0 | -0.85291 | NA | -0.85291 | NA | NA | NA | NA |
| hfEMS:PEMF | 0 | -1.91163 | NA | -1.91163 | NA | NA | NA | NA |
| hfEMS:SEMF | 0 | -1.80118 | NA | -1.80118 | NA | NA | NA | NA |
| hfEMS:TENS | 1 | -0.22157 | -0.22157 | NA | NA | NA | NA | NA |
| HFrTMSC3:M1tDCS | 0 | 0.359386 | NA | 0.359386 | NA | NA | NA | NA |
| HFrTMSC3:PEMF | 0 | -0.69933 | NA | -0.69933 | NA | NA | NA | NA |
| HFrTMSC3:SEMF | 0 | -0.58889 | NA | -0.58889 | NA | NA | NA | NA |
| HFrTMSC3:TENS | 0 | 0.99073 | NA | 0.99073 | NA | NA | NA | NA |
| M1tDCS:PEMF | 0 | -1.05872 | NA | -1.05872 | NA | NA | NA | NA |
| M1tDCS:SEMF | 0 | -0.94827 | NA | -0.94827 | NA | NA | NA | NA |
| M1tDCS:TENS | 0 | 0.631343 | NA | 0.631343 | NA | NA | NA | NA |
| PEMF:SEMF | 0 | 0.110444 | NA | 0.110444 | NA | NA | NA | NA |
| PEMF:TENS | 0 | 1.690061 | NA | 1.690061 | NA | NA | NA | NA |
| SEMF:TENS | 0 | 1.579617 | NA | 1.579617 | NA | NA | NA | NA |

*Abbreviation: 95%CIs: 95% confidence intervals; DLPFCtDCS: anodal over F3 and cathodal over Fp2; drTMS: deep rTMS over bilateral parietal lobe; FREMS: frequency-modulated electromagnetic neural stimulation; hfEMS: high-frequency external muscle stimulation; HFrTMSC3: high frequency rTMS over C3; M1tDCS: anodal over C3 and cathodal over Fp2; NA: not available; NMA: network meta-analysis; OR: odds ratio; PEMF: pulsed electromagnetic fields; RCT: randomized controlled trial; rTMS: repetitive transcranial magnetic stimulation; SEMF: static electromagnetic field; SMD: standardized mean difference; tDCS: transcranial direct current stimulation; TENS: transcutaneous electrical nerve stimulation*

**Reference list of supplement tables:**

1. Page MJ, McKenzie JE, Bossuyt PM, Boutron I, Hoffmann TC, Mulrow CD, et al. The PRISMA 2020 statement: an updated guideline for reporting systematic reviews. Bmj. 2021; 372:n71.

2. You S, Zhang Q, Anitha M, Jia D, Olson DE, Srinivasan S, et al. [Effects of hepatic insulin gene therapy on enteric neuropathy in STZ-diabetic mice]. Zhong Nan Da Xue Xue Bao Yi Xue Ban. 2011; 36(6):546-53.

3. Upton GA, Tinley P, Al-Aubaidy H, Crawford R. The influence of transcutaneous electrical nerve stimulation parameters on the level of pain perceived by participants with painful diabetic neuropathy: A crossover study. Diabetes Metab Syndr. 2017; 11(2):113-8.

4. Jhandi SK, Sharma N, Goyal M. Letter to the editor - Transcranial direct current stimulation improves quality of life and physical fitness in diabetic polyneuropathy: a pilot double blind randomized controlled trial". J Diabetes Metab Disord. 2020; 19(2):2025-6.

5. Szymborska-Kajanek A, Strzelczyk JK, Karasek D, Rawwash HA, Biniszkiewicz T, Cieslar G, et al. Impact of low-frequency pulsed magnetic fields on defensin and CRP concentrations in patients with painful diabetic polyneuropathy and in healthy subjects. Electromagn Biol Med. 2010; 29(1-2):19-25.

6. Klassen A, Di Iorio B, Guastaferro P, Bahner U, Heidland A, De Santo N. High-tone external muscle stimulation in end-stage renal disease: effects on symptomatic diabetic and uremic peripheral neuropathy. Journal of renal nutrition : the official journal of the Council on Renal Nutrition of the National Kidney Foundation. 2008; 18(1):46-51.

7. Graak V, Chaudhary S, Bal BS, Sandhu JS. Evaluation of the efficacy of pulsed electromagnetic field in the management of patients with diabetic polyneuropathy. Int J Diabetes Dev Ctries. 2009; 29(2):56-61.

8. Dufka FL, Munch T, Dworkin RH, Rowbotham MC. Results availability for analgesic device, complex regional pain syndrome, and post-stroke pain trials: comparing the RReADS, RReACT, and RReMiT databases. Pain. 2015; 156(1):72-80.

9. Humpert PM, Morcos M, Oikonomou D, Schaefer K, Hamann A, Bierhaus A, et al. External electric muscle stimulation improves burning sensations and sleeping disturbances in patients with type 2 diabetes and symptomatic neuropathy. Pain medicine. 2009; 10(2):413-9.

10. Julka IS, Alvaro M, Kumar D. Beneficial effects of electrical stimulation on neuropathic symptoms in diabetes patients. J Foot Ankle Surg. 1998; 37(3):191-4.

11. Kornum DS, Bertoli D, Kufaishi H, Wegeberg AM, Okdahl T, Mark EB, et al. Transcutaneous vagal nerve stimulation for treating gastrointestinal symptoms in individuals with diabetes: a randomised, double-blind, sham-controlled, multicentre trial. Diabetologia. 2024; 67(6):1122-37.

12. de Venecia ABF, 3rd, Fresnoza SM. Visual Cortex Transcranial Direct Current Stimulation for Proliferative Diabetic Retinopathy Patients: A Double-Blinded Randomized Exploratory Trial. Brain Sci. 2021; 11(2).

13. Sang P, Zhao J, Yang H. The efficacy of electroacupuncture in among early diabetic patients with lower limb arteriosclerotic wounds. Int Wound J. 2024; 21(4):e14526.

14. Slangen R, Schaper NC, Faber CG, Joosten EA, Dirksen CD, van Dongen RT, et al. Spinal cord stimulation and pain relief in painful diabetic peripheral neuropathy: a prospective two-center randomized controlled trial. Diabetes Care. 2014; 37(11):3016-24.

15. de Vos CC, Meier K, Zaalberg PB, Nijhuis HJ, Duyvendak W, Vesper J, et al. Spinal cord stimulation in patients with painful diabetic neuropathy: a multicentre randomized clinical trial. Pain. 2014; 155(11):2426-31.

16. Aksu S, Hasirci Bayir BR, Sayman C, Soyata AZ, Boz G, Karamursel S. Working memory improvement after transcranial direct current stimulation paired with working memory training in diabetic peripheral neuropathy. Appl Neuropsychol Adult. 2023:1-14.

17. Saadat Z, Rojhani-Shirazi Z, Abbasi L. Dose postural control improve following application of transcutaneous electrical nerve stimulation in diabetic peripheral neuropathic patients? A randomized placebo control trial. Diabetes Metab Syndr. 2017; 11 Suppl 2:S755-S7.

18. Kwan RL, Wong WC, Yip SL, Chan KL, Zheng YP, Cheing GL. Pulsed electromagnetic field therapy promotes healing and microcirculation of chronic diabetic foot ulcers: a pilot study. Adv Skin Wound Care. 2015; 28(5):212-9.

19. Najafi B, Crews RT, Wrobel JS. A novel plantar stimulation technology for improving protective sensation and postural control in patients with diabetic peripheral neuropathy: a double-blinded, randomized study. Gerontology. 2013; 59(5):473-80.

20. Thakkar B, Peterson CL, Acevedo EO. Single Session Effects of Prolonged Continuous Theta Burst Stimulation Targeting Two Brain Regions on Pain Perception in Patients with Painful Diabetic Neuropathy: A Preliminary Study. J Integr Neurosci. 2024; 23(3):54.

21. Thakkar B, Peterson CL, Acevedo EO. Prolonged continuous theta burst stimulation increases motor corticospinal excitability and intracortical inhibition in patients with neuropathic pain: An exploratory, single-blinded, randomized controlled trial. Neurophysiol Clin. 2023; 53(4):102894.

22. Liampas A, Rekatsina M, Vadalouca A, Paladini A, Varrassi G, Zis P. Non-Pharmacological Management of Painful Peripheral Neuropathies: A Systematic Review. Adv Ther. 2020; 37(10):4096-106.

23. Moisset X, Bouhassira D, Avez Couturier J, Alchaar H, Conradi S, Delmotte MH, et al. Pharmacological and non-pharmacological treatments for neuropathic pain: Systematic review and French recommendations. Revue neurologique. 2020; 176(5):325-52.

24. Dosenovic S, Jelicic Kadic A, Miljanovic M, Biocic M, Boric K, Cavar M, et al. Interventions for Neuropathic Pain: An Overview of Systematic Reviews. Anesth Analg. 2017; 125(2):643-52.

25. Pieber K, Herceg M, Paternostro-Sluga T. Electrotherapy for the treatment of painful diabetic peripheral neuropathy: a review. J Rehabil Med. 2010; 42(4):289-95.

26. Kumar D, Alvaro MS, Julka IS, Marshall HJ. Diabetic peripheral neuropathy. Effectiveness of electrotherapy and amitriptyline for symptomatic relief. Diabetes Care. 1998; 21(8):1322-5.

27. Thakur K, Goyal M. Effectiveness of neuromuscular taping on balance, proprioception, pain, and nerve conduction parameters in patients with diabetic peripheral neuropathy: a two-group pretest-posttest randomized sham-controlled trial study protocol. J Diabetes Metab Disord. 2023; 22(2):1793-800.

28. Okdahl T, Bertoli D, Brock B, Krogh K, Knop FK, Brock C, et al. Study protocol for a multicentre, randomised, parallel group, sham-controlled clinical trial investigating the effect of transcutaneous vagal nerve stimulation on gastrointestinal symptoms in people with diabetes complicated with diabetic autonomic neuropathy: the DAN-VNS Study. BMJ Open. 2021; 11(1):e038677.
